# Use of psychotropic medications among glioma patients in Denmark, Norway, Sweden, and Wales

**DOI:** 10.1007/s11060-025-04996-0

**Published:** 2025-04-10

**Authors:** Sarah M. Baxter, Tone Bjørge, Rolf Bjerkvig, Christopher Cardwell, Anders Engeland, Julia Eriksson, Laurel Habel, Jannicke Igland, Kari Klungsøyr, Astrid Lunde, Hrvoje Miletic, Morten Olesen, Anton Pottegård, Johan Reutfors, Mohammad Jalil Sharifian, Marie Linder, Blánaid Hicks

**Affiliations:** 1 Centre for Public Health, Queen’s University Belfast, Belfast, BT12 6BA Northern Ireland; 2 Department of Global Public Health and Primary Care, University of Bergen, N-5020 Bergen, Norway; 3 Cancer Registry of Norway, Norwegian Institute of Public Health, NO-0304 Oslo, Norway; 4 Department of Biomedicine, University of Bergen, N-5020 Bergen, Norway; 5 Department of Chronic Diseases, Norwegian Institute of Public Health, N-0213 Oslo, Norway; 6 Centre for Pharmacoepidemiology, Karolinska Institutet, 171 76 Stockholm, Sweden; 7 Division of Research, Kaiser Permanente Northern California, Oakland, CA 94588 USA; 8 Department of Health and Caring Sciences, Western Norway University of Applied Sciences, 5020 Bergen, Norway; 9 Division of Mental and Physical Health, Norwegian Institute of Public Health, N-0213 Oslo, Norway; 10 Clinical Pharmacology, Pharmacy and Environmental Medicine, Department of Public Health, University of Southern Denmark, 5230 Odense, Denmark

**Keywords:** Glioma, Psychotropics, Brain tumor, Anticonvulsants, Hypnotics

## Abstract

**Purpose:**

Glioma patients often suffer from psychiatric and neurological conditions. However, little is known about the patterns of use of psychotropic drugs pre- and post-glioma diagnosis. Therefore, we assessed temporal patterns of psychotropic prescriptions among glioma patients, compared to an age and sex matched comparison cohort in four European countries.

**Methods:**

Incident gliomas were identified in Wales from the Secured Anonymized Information Linkage Databank (2005–2016) and population-based registries in Denmark (2001–2016), Norway (2006–2019), and Sweden (2008–2018). From each data source, a cancer-free comparison cohort was matched to the glioma cases by age and sex. We calculated rates of new psychotropic prescriptions and any psychotropic prescriptions during the 2 years prior to and post glioma diagnosis. Analyses were stratified by histological subtypes and subclasses of psychotropic medications.

**Results:**

We identified 16,007 glioma patients. The rate of new psychotropic drug use increased from 7 months before diagnosis, peaking around the month of glioma diagnosis (with peak rates ranging from 227 to 753 new psychotropic drugs per 1000 person-months). New use remained substantially higher among glioma patients than comparators throughout the 2-year follow-up period after glioma diagnosis, though rates of new use continued to decline throughout. New use was largely driven by antiepileptics, anxiolytics, hypnotics, and sedatives. Patterns were similar when analyses were stratified by histological subtype.

**Conclusion:**

Psychotropic drug use among glioma patients was high, and elevations observed around the time of cancer diagnosis, largely driven by antiepileptics, anxiolytics, hypnotics, and sedatives, are likely associated with the consequences of the disease.

**Supplementary Information:**

The online version contains supplementary material available at 10.1007/s11060-025-04996-0.

## Introduction

Glioma, the most common type of malignant brain tumor, continues to cause significant morbidity and mortality worldwide due to limited treatments available [1, 2]. Common presenting symptoms of glioma include cognitive changes, seizures, and motor dysfunction, which may change throughout the disease trajectory [3–6].

Although psychiatric (e.g., depression) and neurological (e.g., epilepsy) comorbidities [6–11] are well documented in glioma patients [12–14], gaps in evidence on psychotropic prescribing still exist. One study found antidepressant use remained twice as high and sedative use three times higher in glioma patients compared to controls 1 year post-diagnosis [12]. However, this study focused on low-grade gliomas, and psychotropic drugs such as antipsychotics or anxiolytics were not considered [12]. Similar limitations exist in other studies, which either focused on limited medication classes or tumor characteristics or lacked information from prescription records [13, 14]. Evaluating psychotropic drug use in the years before a glioma diagnosis may provide insights into the early presentation of the cancer, as pre-diagnosis prescribing may reflect symptom relief strategies.

Understanding changes in psychotropic drug use around glioma diagnosis is important. Preclinical evidence suggests that medications like antidepressants, antipsychotics, and antiepileptics—which cross the blood–brain barrier—may have antineoplastic effects [15–17]. New drug use may reflect glioma symptoms, surveillance, or comorbidities identified during diagnosis, while discontinuation often occurs in end-of-life care. These factors complicate epidemiological studies by introducing confounding and reverse causality. [18]. A comprehensive assessment of psychotropic drug use is therefore essential to inform future epidemiology research in this area.

Given the lack of evidence on prescribing trends of psychotropic medications among glioma patients, we aimed to examine the prescribing patterns of psychotropic medication around glioma diagnosis with a focus on glioma subtypes and individual psychotropic classes.

## Materials and methods

### Data sources

This multinational drug utilization study used a common protocol and shared syntax with some necessary country-specific differences to examine the use of psychotropic medications in glioma patients using data from nationwide population-based registries in Denmark, Norway, and Sweden and the Secure Anonymized Information Linkage (SAIL) Databank in Wales [19–22]. Detailed information on each data source is provided in Supplementary Information 1.

The nationwide registries in Denmark, Norway, and Sweden hold information for the entire population on demographic parameters and health data, including cancer diagnoses and prescription medication use [19]. The national prescription registries contain data on all prescription drugs dispensed to residents in the community, including the type of drug, the date, and quantity dispensed. Medications are categorized according to the World Health Organization Anatomic Therapeutic Chemical (ATC) index [23]. National cancer registries provide accurate and complete registrations of incident cancers in each nation. Cancer diagnoses are recorded using the International Classification of Diseases, version 10 (ICD-10), and the ICD for Oncology (ICD-O-3) for details on topography and morphology. These are linked to population registries through a unique personal identifier, providing details of births, migrations, and deaths.

The SAIL Databank is a population-based data repository that can link datasets based on an encrypted identifier [20, 22]. The Welsh Longitudinal General Practice Dataset contains primary care records of approximately 80% of the Welsh population and includes medications prescribed (coded via the National Health Service Read Code version 2 [24]). The quantity prescribed is not available. The Welsh Cancer Intelligent Unit dataset (National Cancer Registry) captures all incident cancer diagnoses (ICD-10 codes) for Welsh residents, and morphology (ICD-O-3 codes). The Annual District Death Extract captures death data.

### Study population

We identified all patients ≥ 18 years (≥ 21 years in Wales) with a primary diagnosis of histologically verified glioma (see Supplementary Table 1 for definition). We excluded children due to the differences in types of gliomas and cancer biology between children and adults. We included individuals diagnosed with glioma between 2005 and 2016 in Wales, 2001–2016 in Denmark, 2006–2019 in Norway, and 2008–2018 in Sweden. We excluded those with a previous cancer diagnosis (except non-melanoma skin cancer) and those with less than 2 years of data availability prior to glioma diagnosis (in Wales at least 3 years of data availability before glioma diagnosis was required due to left truncation). The date of glioma diagnosis was considered the index date. For each glioma patient, we randomly selected up to 10 risk-set comparators who had no history of glioma at the index date and were matched on sex, age, general practitioner (GP) practice (Wales), and year data coverage began [25]. The same exclusion criteria were applied to comparators as for cases. Comparators were assigned an index date identical to the diagnosis date for their matched case. All individuals were followed from two years before their index date until the date of death, migration, end of coverage, or 2 years after their index date, whichever occurred first.

### Study drugs

We evaluated all psychotropic medications, including antidepressants, antipsychotics, anxiolytics, anti-epileptics, hypnotics and sedatives, psychostimulants, and antihistamines (based on the ATC classification; Supplementary Table 1). Psychotropic prescriptions were determined from prescribing records from GP practices in Wales and filled prescriptions (dispensing) records from pharmacies in Denmark, Norway, and Sweden. Any (all) prescription use was defined as the total number of psychotropic prescriptions within a 1-month period. New (incident) prescription use was defined as a prescription for a psychotropic medication, with no previous prescription for psychotropic medications during the 2 years prior. Therefore, to determine new use, a 4-year baseline period (which included a 2-year wash-out and a 2-year exposure period) was required before glioma diagnosis. Thus, only individuals with a 4-year look-back period were included in the analysis of new use.

### Statistical analyses

Categorical variables were presented as counts and percentages and continuous variables as medians with interquartile ranges (IQR). In analysis of both new and any use, we examined psychotropic drug use from 2 years before to 2 years after the index date in 1-month intervals. We defined 1 month as 30 days, counting forward in 30-day intervals from 2 years prior to index date to 2 years after index date. Time zero was anchored at the index date in both cohorts. First, we calculated rates of new and any psychotropic drug use, estimating monthly rates per 1000 person-months, i.e., the number of either new or total prescriptions were counted for each 1-month interval during the 4 years of follow-up and divided it by the number of person-months in that interval to estimate the rate. Analyses were completed for all psychotropic medications combined and for individual drug classes (defined in Supplementary Table 1). In analyses of individual drug classes, new prescription use was defined as a prescription within a specific drug class with no previous prescription within the same drug class in the 2 years prior to the prescription in question. Any (all) prescription use was defined as the total number of prescriptions within a specific drug class in a specified interval.

Second, we described the proportion of individuals in both cohorts using psychotropic drug classes during the period where the incidence rate of psychotropic medication was highest for glioma patients. Similarly, we described the proportion of glioma patients using each psychotropic drug class in 6-month intervals from 24 months before to 24 months after glioma diagnosis. Use of a psychotropic drug class was considered as at least one prescription within the 6-month interval. Finally, we described the volume of drug used of the most common drug classes by examining the defined daily doses (DDDs) per 1000 person-months, 24 months before and after the index date (not available in Wales).

Analyses were repeated stratified by histological glioma subtype. Glioma subtypes were based on a modified 2007 WHO classification of tumors of the Central Nervous System and were classified by a neuropathologist (HM) (Supplementary Table 1). Due to small numbers, histological subtypes were collapsed into three categories in Norway and Wales (glioblastoma, diffuse astrocytoma, and other category). We used SAS v9.4 (SAS Institute, North Carolina, USA), SPSS v29 (IBM, New York, USA), and Stata v18 (StataCorp, Texas, USA) for data management and analysis.

### Ethical Approval

Study approval was granted by the Danish Health Data Authority, the Information Governance Review Panel in Wales, the Regional Committee for Medical and Health Research Ethics in Norway (2018/2125/REK vest), and the Swedish Ethical Authority.

## Results

### Characteristics of the study population

Overall, 16,007 glioma patients and 141,312 matched comparators were included. Most glioma cases were glioblastoma multiforme (≥ 60%) (Table 1). Across all countries, there were more males with glioma than females (60% vs 40%). The median age at diagnosis was higher in Wales at 65 years (IQR 55–73) than in Denmark at 61 years (IQR 50–69), Norway at 60 years (IQR 48–70), and Sweden at 60 years (IQR 48–69) (Table 1).

**Table 1:** Characteristics of glioma patients by country

Characteristics	Denmark	Norway	Sweden	Wales
Number of patients	4,942	4,210	5,361	1,494
Age (years), Median (IQR)	61 (50–69)	60 (48–70)	60.5 (49–69)	65 (55–73)
Sex (n, %)				
Male	2,924 (59%)	2,489 (59%)	3,260 (61%)	890 (60%)
Female	2,018 (41%)	1,721 (41%)	2,101 (39%)	604 (40%)
Glioma subtypes (n, %)				
Glioblastoma	3,261 (66%)	2,610 (62%)	3,499 (65%)	942 (63%)
Diffuse astrocytoma	743 (15%)	541 (13%)	810 (15%)	134 (9%)
Oligodendroglioma	579 (12%)	281 (7%)	469 (9%)	79 (5%)
Oligoastrocytoma	42 (1%)	151 (3%)	182 (3%)	19 (1%)
Other	317 (6%)	627 (15%)	401 (8%)	320 (22%)

### New prescription use

**Fig. 1:**
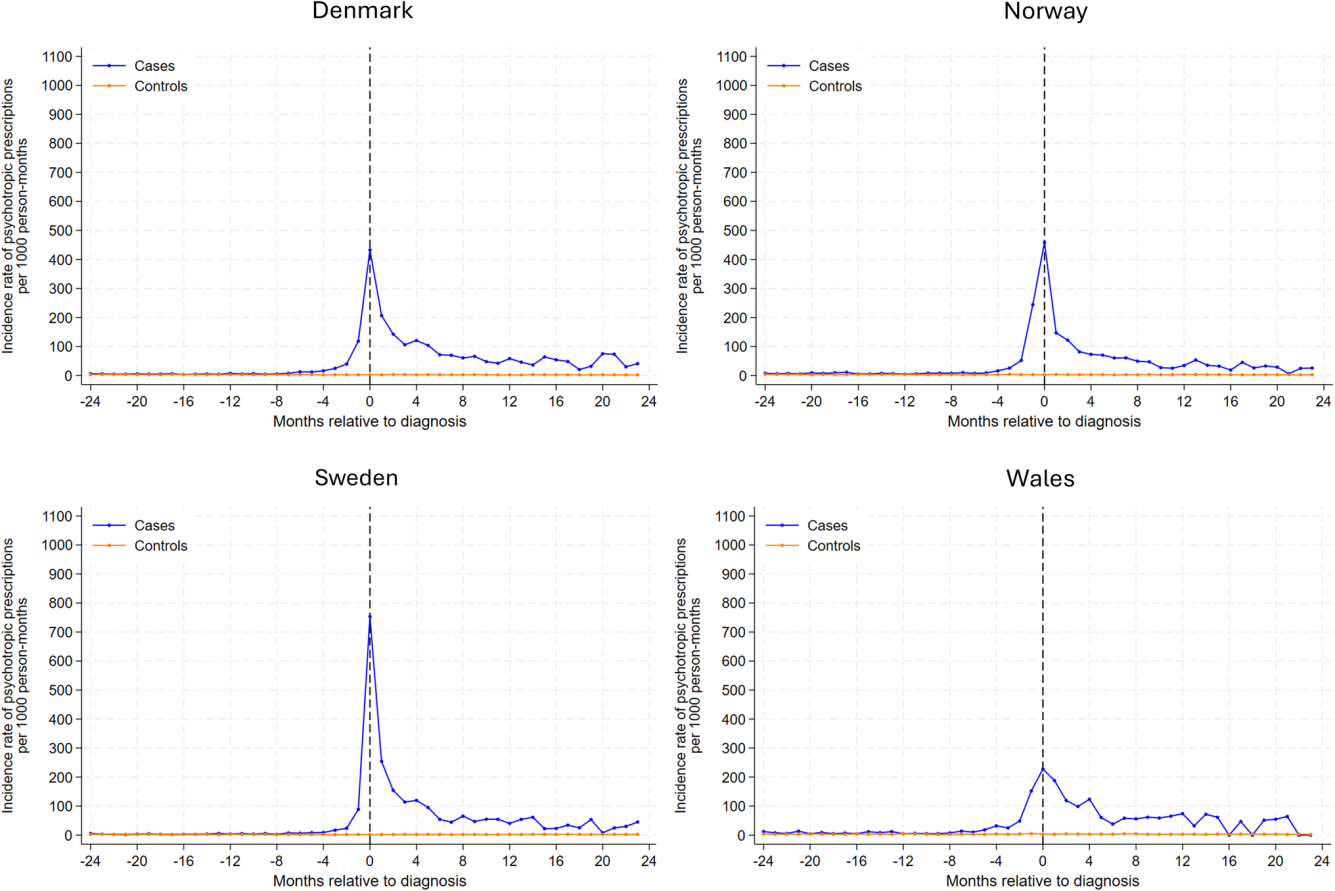
Rates of new psychotropic prescriptions in 1-month intervals before and after the month of glioma diagnosis among glioma patients and the comparison cohort matched on age, sex and year coverage began by study site

Analysis of new psychotropic drug use (Fig. 1) found similar rates for glioma and comparison cohorts until 5–7 months before index date across all countries. After this time, the rate of new psychotropic drug use increased considerably in glioma patients, peaking at the month of diagnosis. The highest incidence rate at month of diagnosis was observed in Sweden (753 prescriptions per 1000 person-months), and the lowest peak incidence rate was observed in Wales (227 prescriptions per 1000 person-months). Following glioma diagnosis, the incidence rate decreased but remained considerably higher among glioma patients than comparators throughout the 2-year post-diagnosis follow-up period. During the period of highest new use (i.e., 7 months before index date and 8 months after index date), we found new use was largely driven by antiepileptics with the proportion of glioma patients receiving antiepileptics during this period, ranging from 35% in Wales to 51% in Sweden, followed by hypnotics and sedatives, and anxiolytics (Table 2).

**Table 2:** Psychotropic drug classes initiated among glioma patients around the time of glioma diagnosis (between 7 months before and 8 months after diagnosis)

Drug class (n, %)	Denmark		Norway		Sweden		Wales	
	Glioma cohort^a^ (4,918)	Comparison cohort^a^ (48,766)	Glioma cohort^a^ (3,586)	Comparison cohort^a^ (32,873)	Glioma cohort^a^ (5,330)	Comparison cohort^a^ (40,098)	Glioma cohort^a^ (1,428)	Comparison cohort^a^ (12,348)
Antiepileptics	2,034 (41%)	490 (1%)	1,504 (42%)	281 (0.9%)	2,736 (51%)	318 (0.8%)	501 (35%)	147 (1%)
Anxiolytics	1,516 (31%)	779 (2%)	1,125 (31%)	646 (2%)	2,209 (41%)	918 (2%)	228 (16%)	210 (2%)
Hypnotics & sedatives	1,399 (28%)	889 (2%)	1,142 (32%)	803 (2%)	1,940 (36%)	918 (2%)	344 (24%)	171 (1%)
Antidepressants	994 (20%)	1,029 (2%)	317 (9%)	616 (2%)	812 (15%)	802 (2%)	191 (13%)	431 (4%)
Antipsychotics	415 (8%)	323 (0.7%)	217 (6%)	279 (0.9%)	216 (4%)	119 (0.3%)	148 (10.4%)	252 (2%)
Psychostimulant	18 (0.4%)	34 (0.1%)	< 5	28 (0.1%)	20 (0.4%)	56 (0.1%)	< 5	< 5
Antihistamines	249 (5%)	1,059 (2%)	287 (8%)	946 (3%)	92 (2%)	241 (0.6%)	288 (20%)	370 (3%)

^a^As the new use analysis required an additional 2-year baseline, the individuals for this analysis were limited to those with 4 years of data availability

Similar trends in new psychotropic drug use were seen in analyses stratified by histological glioma subtypes (Supplementary Figure 2). The highest new psychotropic drug use around the time of diagnosis was observed for diffuse astrocytoma, followed by oligoastrocytoma and oligodendroglioma.

**Fig. 2:**
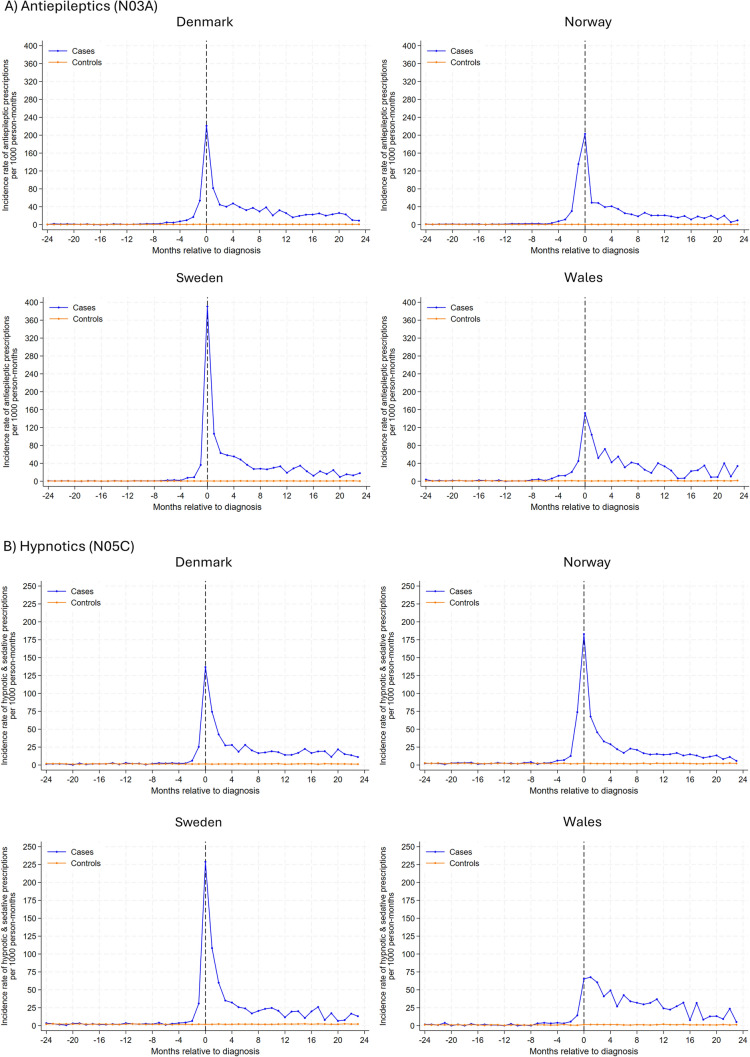

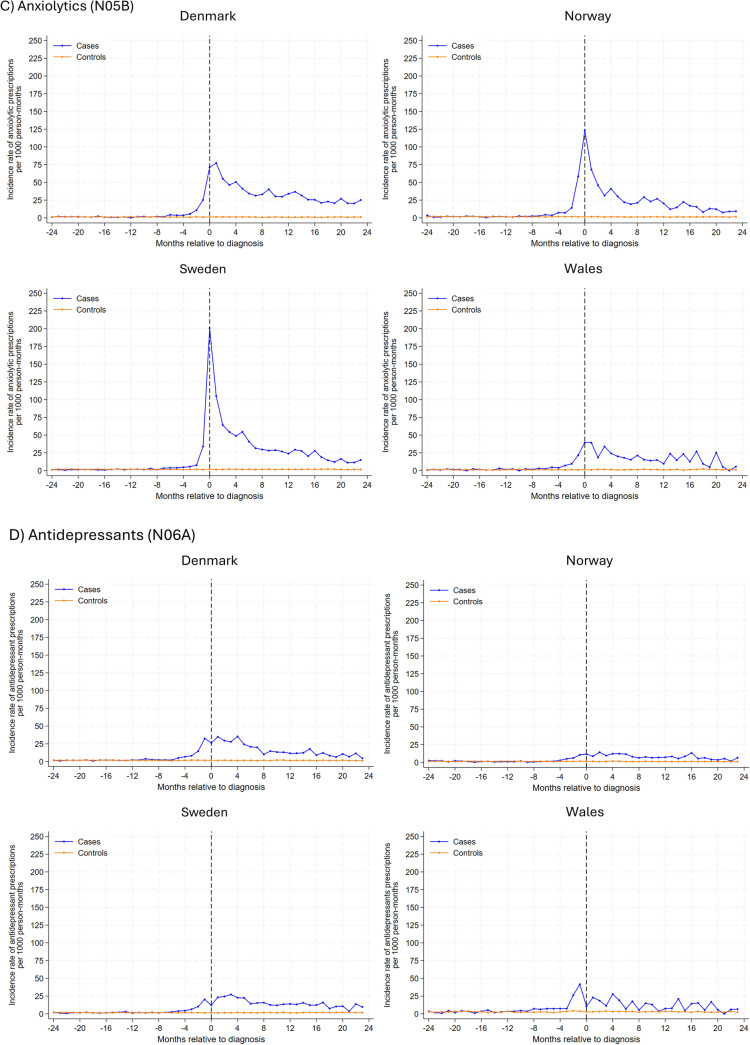

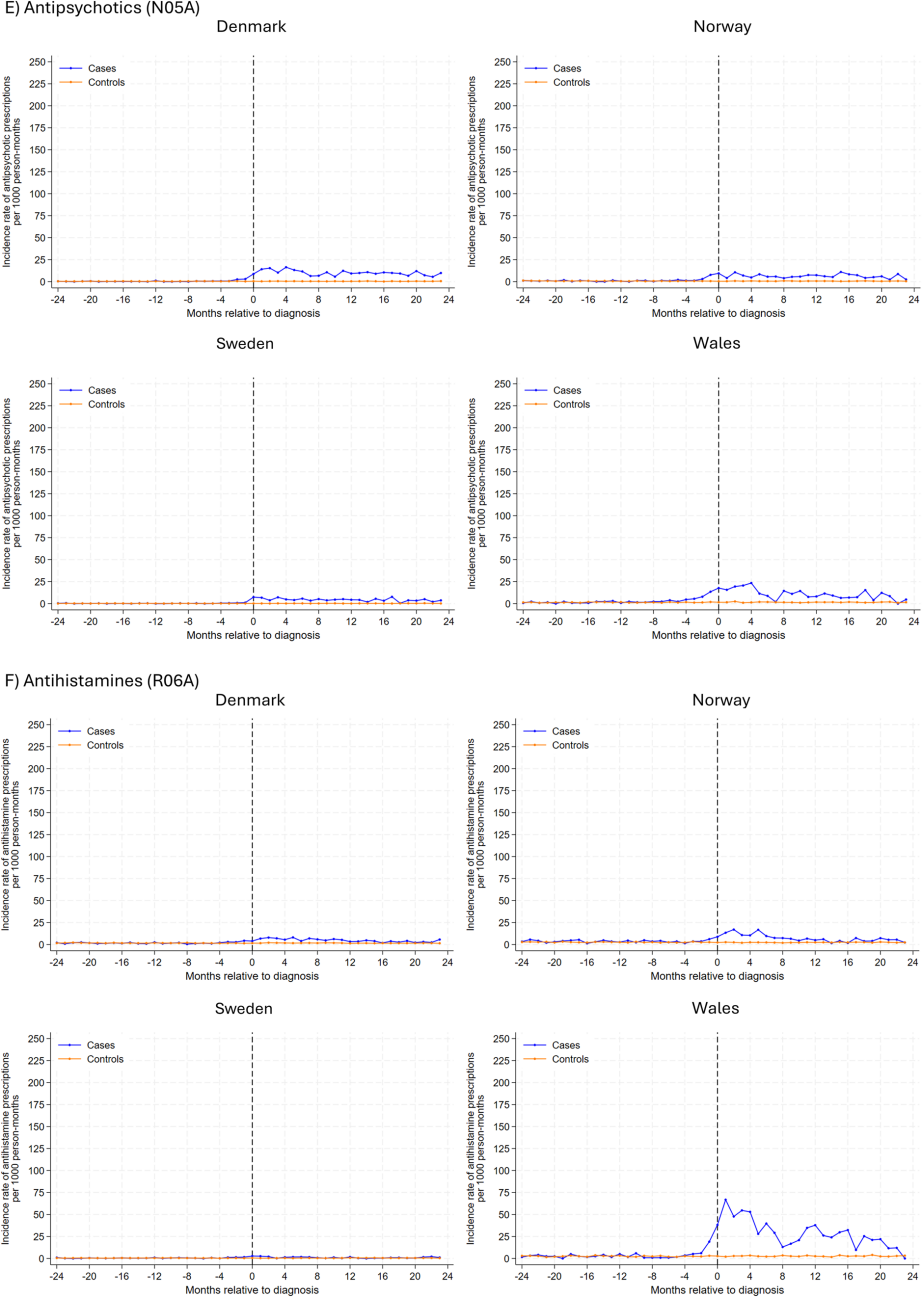

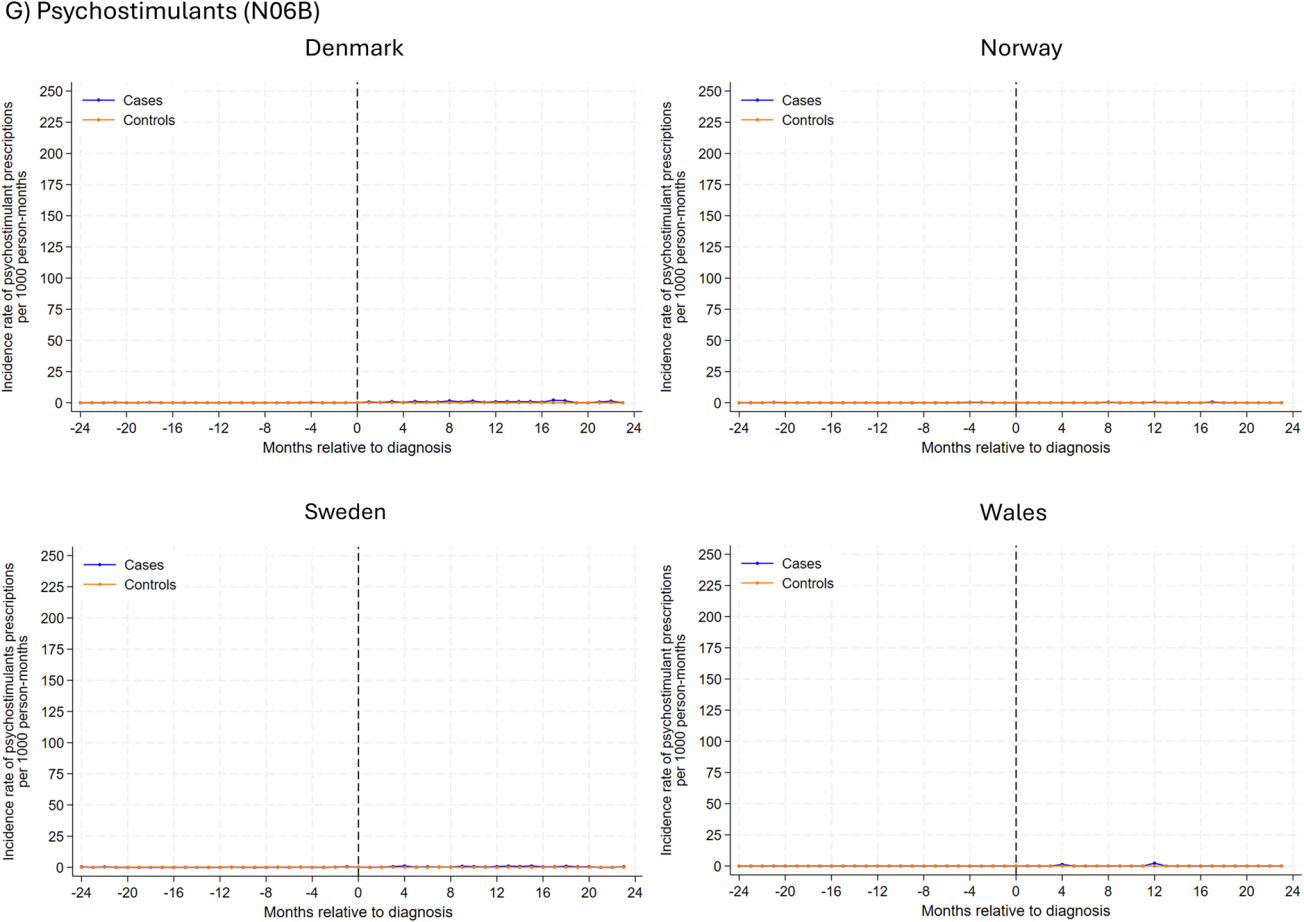
Rates of new prescriptions in 1-month intervals before and after month of glioma diagnosis among glioma patients and the comparison cohort matched on age, sex and year coverage began by psychotropic drug class and study site. Note different y axis

In analysis of individual drug classes (Fig. 2), antiepileptic drugs had the highest rate of new use among glioma patients, with the highest rates observed at the month of diagnosis (ranging from 391 prescriptions per 1000 person-months in Sweden and 152 in Wales). During the 2-year follow-up post-diagnosis, rates decreased but remained somewhat higher than comparators. Similar trends were observed for anxiolytics, hypnotics, and sedatives, albeit overall rates were lower. In Denmark, Sweden, and Norway, incident antidepressant use peaked 2–4 months after the index date, gradually decreasing thereafter. In Wales, the incidence rate for antidepressants peaked 1-month before diagnosis, with a subsequent gradual decline post-diagnosis. A small peak was observed for new antipsychotic use 2–4 months post-diagnosis for glioma patients, and the rate remained largely similar hereafter. Generally, incidence rates for use of psychostimulants and antihistamines were similar between glioma patients and their matched comparators, except in Wales where a small peak in antihistamine use was observed for glioma patients after diagnosis .

### Any psychotropic drug use

In analysis of any psychotropic drug use, we observed a sharp increase in psychotropic drugs among glioma patients from 4 months before diagnosis, with rates remaining considerably higher among cases than comparators for the duration of the follow-up period (Supplementary Figure 3). In analysis stratified by histological glioma subtypes, trends were similar to the new use analysis (Supplementary Figure 4).

Similar findings were observed when stratified by drug class. Different patterns of use were observed between the drug classes (Supplementary Figure 5). A sharp increase in antiepileptic use was observed at diagnosis, with increasing use throughout the 2-year follow-up period, peaking around 12–18 months. Hypnotic and anxiolytic use generally peaked around diagnosis with a gradual decline in the post-diagnosis period, although in Wales, there was a more gradual increase with rates peaking around 12 months post-diagnosis. Any antidepressant use was higher among glioma patients than in comparators, with elevated use peaking at the time of diagnosis or shortly after. Use of antipsychotics increased shortly before or after glioma diagnosis, and use remained somewhat higher during the 2-year post-diagnosis period. Generally,  the rates of any psychostimulant and antihistamine use were similar between glioma patients and their comparators.

Analysis evaluating the prevalence of glioma patients with at least one prescription for a specific psychotropic class in 6-month intervals from 24 months before diagnosis to 24 months after diagnosis found increases in the prevalence of patients receiving prescriptions for antiepileptics, anxiolytics, hypnotics, and sedatives (Table 3). Most drug classes displayed a peak after diagnosis, and use remained high in subsequent time intervals. For example, the prevalence of glioma patients using antiepileptics increased from approximately 3% in the 18–24 months before diagnosis to around 40% in the first 6 months after diagnosis, remaining around 50% in the 18–24 months after diagnosis. In contrast, small increases around the time of diagnosis were observed for antidepressants and antipsychotics, with a slight decline thereafter.

**Table 3:** Prevalence of psychotropic drug use by medication class in 6 months intervals from 24 months before to 24 months post glioma diagnosis

Drug class* n (%)*	Months prior to glioma diagnosis				Months after glioma diagnosis			
	24–18	18–12	12–6	6–0	0–6	6–12	12–18	18–24
Antiepileptics								
Denmark	167 (3.4%)	176 (3.6%)	206 (4.2%)	615 (12%)	2,068 (42%)	1,846 (51%)	1,417 (53%)	1,067 (55%)
Norway	145 (3.4%)	155 (3.7%)	184 (4.4%)	829 (20%)	1,801 (43%)	1,571 (48%)	1,219 (49%)	974 (50%)
Sweden	132 (2.5%)	138 (2.6%)	157 (2.9%)	414 (7.7%)	2,773 (52%)	2,398 (56%)	1,761 (55%)	1,319 (55%)
Wales	80 (5.4%)	85 (5.7%)	93 (6.2%)	219 (15%)	552 (37%)	393 (54%)	272 (56%)	212 (62%)
Hypnotics & sedatives								
Denmark	252 (5.1%)	254 (5.1%)	274 (5.5%)	424 (8.6%)	1,550 (31%)	823 (22%)	531 (20%)	360 (19%)
Norway	392 (9.3%)	406 (9.6%)	429 (10%)	756 (18%)	1,553 (36%)	860 (26%)	583 (23%)	412 (21%)
Sweden	381 (7.1%)	387 (7.2%)	428 (8.0%)	608 (11%)	2,297 (43%)	1,209 (28%)	798 (25%)	548 (23%)
Wales	64 (4.3%)	60 (4%)	53 (3.5%)	100 (6.7%)	364 (24.4%)	189 (26%)	124 (26%)	51 (15%)
Anxiolytics								
Denmark	223 (4.5%)	220 (4.5%)	220 (4.5%)	438 (8.9%)	1,520 (31%)	981 (27%)	653 (25%)	398 (21%)
Norway	245 (5.8%)	249 (5.9%)	263 (6.2%)	591 (14%)	1,352 (32%)	798 (24%)	508 (20%)	338 (17%)
Sweden	183 (3.4%)	198 (3.7%)	222 (4.1%)	456 (8.5%)	2,267 (42%)	1,230 (28%)	775 (24%)	472 (20%)
Wales	41 (2.7%)	42 (2.8%)	51 (3.4%)	105 (7%)	211 (14%)	116 (16%)	87 (18%)	51 (15%)
Antidepressants								
Denmark	331 (6.3%)	328 (6.6%)	368 (7.4%)	645 (13%)	1,078 (22%)	807 (22%)	556 (21%)	367 (19%)
Norway	299 (7.1%)	307 (7.3%)	303 (7.2%)	396 (9.4%)	463 (11%)	404 (12%)	308 (12%)	225 (12%)
Sweden	417 (7.8%)	442 (8.2%)	432 (8.1%)	647 (12%)	1,029 (19%)	846 (20%)	647 (20%)	477 (20%)
Wales	173 (12%)	184 (12%)	200 (13%)	308 (21%)	223 (15%)	134 (18%)	96 (20%)	75 (22%)
Antipsychotics								
Denmark	71 (1.4%)	70 (1.4%)	72 (1.5%)	106 (2.1%)	398 (8.1%)	260 (7.1%)	203 (7.6%)	131 (6.8%)
Norway	90 (2.1%)	89 (2.1%)	89 (2.1%)	141 (3.3%)	231 (5.5%)	172 (5.2%)	141 (5.6%)	91 (4.7%)
Sweden	63 (1.2%)	65 (1.2%)	68 (1.3%)	83 (1.5%)	230 (4.3%)	176 (4.1%)	122 (3.8%)	74 (3.1%)
Wales	33 (2.2%)	33 (2.2%)	34 (2.3%)	82 (5.5%)	126 (8.4%)	60 (8.2%)	41 (8.4%)	21 (6.2%)
Psychostimulants								
Denmark	5 (0.1%)	5 (0.1%)	< 5	5 (0.1%)	15 (0.3%)	21 (0.6%)	18 (0.7%)	13 (0.7%)
Norway	6 (0.1%)	5 (0.1%)	6 (0.1%)	8 (0.2%)	6 (0.1%)	8 (0.2%)	8 (0.3%)	6 (0.3%)
Sweden	17 (0.3%)	14 (0.3%)	19 (0.4%)	21 (0.4%)	24 (0.4%)	24 (0.6%)	27 (0.8%)	20 (0.8%)
Wales	< 5	< 5	< 5	< 5	< 5	< 5	< 5	< 5
Antihistamines								
Denmark	130 (2.6%)	133 (2.7%)	129 (2.6%)	159 (3.2%)	222 (4.5%)	163 (4.5%)	107 (4.0%)	67 (3.5%)
Norway	365 (8.7%)	403 (9.6%)	416 (9.9%)	422 (10%)	498 (12%)	352 (11%)	259 (10%)	210 (11%)
Sweden	35 (0.7%)	40 (0.7%)	56 (1.0%)	55 (1.0%)	87 (1.6%)	48 (1.1%)	31 (1.0%)	31 (1.3%)
Wales	74 (5%)	82 (5.5%)	85 (5.7%)	115 (7.7%)	324 (22%)	153 (21%)	100 (21%)	56 (17%)

In analyses of the volume of drugs filled, trends were generally comparable to the any use analysis (Supplementary Figure 6). In sensitivity analysis, similar findings were observed for both the new use and any use analysis when restricting glioma diagnoses to those in 2008–2016 (Supplementary Figures 7, 8).

## Discussion

In this multinational study, we observed a substantial increase in psychotropic medication use among glioma patients within 5–7 months before diagnosis. A peak in new prescriptions, driven largely by antiepileptics likely indicated for underlying glioma symptoms, was seen around diagnosis. The rate of new psychotropic prescriptions remained elevated among glioma patients compared to the comparison cohort in the 2 years post-diagnosis.

Although psychotropic drug use around glioma diagnosis was similar between the four countries, some variation was observed. Sweden has the highest incidence, while Wales had the lowest rates. Disparities may stem from differences in how prescription data is captured (e.g., GP clinical systems in Wales vs. prescription registries in Nordic countries), prescribing differences between countries, or a lower threshold to initiate psychotropic medications. For example, anxiolytic and antidepressant use in the general population is higher in Sweden than in Norway and Denmark. [26]. Furthermore, the UK general population uses fewer anxiolytics compared to Nordic countries, indicating that prescribing practices may differ, however, specific evidence for a glioma population is lacking [26, 27].

Our findings have clinical implications. Around the time of glioma diagnosis, there was a considerable increase in new psychotropic drugs, driven by drugs to manage glioma symptoms (e.g., antiepileptics) or psychological effects of the establishment of disease (e.g., anxiolytics, hypnotics, sedatives). Post-diagnosis, approximately 50% of glioma patients used anti-epileptics, reflecting the common occurrence of epilepsy in glioma, and treatment with antiepileptics is often important to reduce further complications and decline in quality of life [11, 28]. We observed increased use of medications used for anxiety, depression, and sleeping disorders, which could be attributable to the disease itself, psychological impact of diagnosis, or treatment side effect [29–31]. These conditions can have a considerable impact on a patient’s quality of life [30, 32, 33] and depression has been associated with poorer overall survival [32]. Therefore, glioma patients must be regularly monitored for and effectively treated for these conditions. However, to date, there is a lack of studies examining effective management of these conditions in glioma patients, particularly for non-pharmacological treatments [30].

Our findings revealed that the use of any anxiolytic, hypnotic, sedatives, and antidepressant remained elevated after diagnosis. Although rates decreased in the 2 years post-diagnosis, they remained elevated at the end of follow-up, indicating the long-term physical and psychological impact that glioma has following diagnosis and into either living with glioma as a chronic condition or survivorship [30, 34]. The decrease in medication use likely indicates patient death or discontinuation of medication, particularly as patients approach end of life. Short-term use aligns with recommendations, and the experience of psychological distress varies throughout the disease [35], therefore, medication may be discontinued as psychological distress subsides [36, 37].

Our findings also have some important implications for future pharmacoepidemiological studies. Psychotropic medication use around glioma diagnosis is not a random event, particularly in the 5–7 months prior. Medication initiated before glioma diagnosis is likely related to diagnostic workup or symptoms of the undiagnosed disease, while those initiated after diagnosis may treat symptoms or the psychological impact of diagnosis. Initiation of medication may be influenced by increased surveillance, co-morbidities, symptom burden, and prognosis. Including medications in the period before diagnosis could lead to spurious associations between psychotropic drug use and glioma risk due to reverse causality [38]. Although prior evidence suggests that a lag of 6 months would be an adequate lag-time to account for reverse causality [18], our findings indicate that a lag-time period of 1-year would be more suitable for glioma, particularly in studies evaluating the potential anti-tumor effects of psychotropic drugs such as antidepressants [16, 17].

A strength of this study was the multinational approach, permitting a comprehensive analysis and comparison of psychotropic medication use among glioma patients. Using a common data model allowed the standardization of glioma subtypes and medication definitions between countries. Data from three nationwide registries were included, minimizing the risk of selection and misclassification bias [39]. However, some limitations need to be considered. The primary weakness of this study was the lack of prescription information from inpatient hospital stays or palliative care, meaning we may have underestimated rates of psychotropic prescribing, as palliative care is common in this population [40]. We lacked information on tumor location or gene mutations, which may impact prescribing, as neurological and psychiatric symptoms may depend on the location of the tumor or tumor phenotype [9, 41]. Furthermore, we lacked information on grading and disease progression, which may impact if a clinician initiates, continues, or deprescribes certain medicines. Prescription data from Wales were based on medications prescribed in general practice rather than dispensed, potentially overestimating prescribing rates. Variations in data availability across countries prevented us from determining the underlying reason for drug prescriptions. This limitation was particularly relevant for drugs with multiple indications, such as antiepileptics, where there have been shifts in the primary indications over time [42]. While for certain indications, such as bipolar affective disorder, prescription rates would be expected to be similar between cases and controls, conditions like generalized anxiety disorder are likely to be more prevalent among cancer patients[43]. Therefore, further research is needed to elucidate the specific indications for psychotropic medication use in glioma patients. Finally, this study used the 2007 WHO classification of tumors of the Central Nervous System. This has subsequently undergone significant revisions in 2016 and 2021, incorporating molecular markers [44, 45]. Unfortunately, we lacked molecular information to undertake retrospective classification. However, given the study period, this reflects the criteria used in the original histopathological diagnoses and the real-world clinical decision-making for patients during the study period. This also allows for comparison with previous studies. Despite this, future studies utilizing current classifications are warranted.

We found that psychotropic medications are used frequently among glioma patients, notably around the time of glioma diagnosis. Associations were largely driven by antiepileptics, anxiolytics, hypnotics, and sedatives and are likely associated with the consequences of disease. Future studies evaluating the underlying indications of psychotropic medications in this population are warranted.

## Supplementary Information

Below is the link to the electronic supplementary material.

Supplementary file1 (PDF 4909 KB)

## Data Availability

In Denmark deidentified data can be made available for authorized researchers after application to Forskerservice at the Danish Health Data Authority (information on application process): https://sundhedsdatastyrelsen.dk/da/forskerservice. In Norway deidentified data can be made available for authorized researchers after application to helsedata.no at the Norwegian Health Data Authority (Helsedataservice). Information on application process: https://helsedata.no/en/access-to-data/. In Sweden deidentified data can be made available to authorized researchers after applying for ethical approval and, if approved, for data extraction from Statistics Sweden and the National Board of Health and Welfare. Information on the application process: •https://etikprovningsmyndigheten.se/for-forskningsperson/ •https://bestalladata.socialstyrelsen.se/data-for-forskning/ •https://www.scb.se/en/services/ordering-data-and-statistics/microdata In Wales, deidentified data can be made available for authorized researchers after application to SAIL Databank. Information on application process: https://saildatabank.com/
