# Supplementary material for: Use of psychotropic medications among glioma patients in Denmark, Norway, Sweden, and Wales
Source: J Neurooncol. 2025 Apr 10;173(2):383–95. doi: 10.1007/s11060-025-04996-0 (PMC12106481; doi:10.1007/s11060-025-04996-0)

Utilization of psychotropic medications among patients diagnosed with glioma: A multinational drug utilisation study

Supplementary Materials

Contents

Supplementary Information 1 Data sources 3

Supplementary Table 1 Definitions of glioma and psychotropic medications 6

Supplementary Table 2 Drugs available in each country during the study period 8

Supplementary Figure 1 Flow chart for inclusion, by study site 12

Supplementary Figure 2 Rates of new psychotropic prescriptions in 1-month intervals before and after the month of glioma diagnosis among glioma patients and age and sex matched comparison cohort by histological subtype and study site 14

Supplementary Figure 3 Rates of all prescriptions for psychotropic medication in 1-month intervals before and after month of glioma diagnosis among glioma patients or among age and sex matched comparison cohort by study site 17

Supplementary Figure 4 Rates of all prescriptions for psychotropic medication in 1-month intervals before and after month of glioma diagnosis among glioma patients or among age and sex matched comparison cohort by histological subtype and study site. 18

Supplementary Figure 5 Rates of all prescriptions for psychotropic medication in 1-month intervals before and after month of glioma diagnosis among glioma patients or among age and sex matched comparison cohort by individual drug class and study site. Note different y axis. 22

Supplementary Figure 6 Rates of DDDs for psychotropic medication in 1-month intervals before and after month of glioma diagnosis among glioma patients or among age and sex matched comparison cohort by individual drug class and study site. Note different y axis. 26

Supplementary Figure 7 Rates of new psychotropic prescriptions in 1-month intervals before and after the month of glioma diagnosis among glioma patients and the age and sex matched comparison cohort by study site restricted to 2008-2016. 29

Supplementary Figure 8 Rates of all prescriptions for psychotropic medication in 1-month intervals before and after month of glioma diagnosis among glioma patients or among age and sex matched comparison cohort by study site restricted to 2008-2016. 30

Supplementary Information 1 Data sources

Danish Nationwide Health Registries

The Danish Cancer Registry has recorded incident cases of cancer on a nationwide basis since 1943 and provides accurate and almost complete records of cancer cases in Denmark. Cancer diagnoses are coded according to the International Classification of Diseases, Tenth Revision 10 (ICD-10) and the ICD for Oncology (ICD-O-1-3) for topography and morphology.

The Danish National Prescription Registry contains data on all prescription drugs filled by Danish residents since 1995. The data include the type of drug, date of filling, and quantity. The dosing information and the indication for prescribing are not available and no information is available on drugs used at hospital level. Drugs are categorized according to the Anatomic Therapeutic Chemical (ATC) index, a hierarchical classification system developed by the WHO, and the quantity dispensed for each prescription is described by the number and strength of the pharmaceutical entities (e.g., tablets), as well as defined daily doses (DDD).

The Danish Civil Registration System contains data on addresses, migration, and date of death. This system allowed us to extract population controls and to keep track of all subjects during the study period.

Norwegian Nationwide Registries

The Cancer Registry of Norway (CRN) was established in 1951, and contains mandatory reports on all new cancer cases and certain pre-cancerous lesions in Norway. Clinical reports, pathology notifications and death certificates provide information about cancer site, histological type and stage of disease at the time of diagnosis. Main cancer types are classified according to ICD-10 (International Classification of Diseases, version 10).

The Norwegian Prescription Database (NorPD) records all prescribed drugs dispensed by pharmacies since January 1st, 2004, except those dispensed to institutionalized patients (e.g. nursing homes, hospitals). The latter are recorded at an institutional level only and hence could not be included in the study. For each prescription in the database, information on age and sex of the patient, dispensing date, and detailed drug information, including amount dispensed in Defined Daily Doses (DDD) and number of packages, are recorded. Drugs are code according to the international Anatomical Therapeutic Chemical (ATC) classification system. Drugs for chronic diseases are reimbursed by the national insurance scheme, with the reimbursement code (based on local codes prior to 2008 and on ICD-10 and ICPC (International Classification of Primary Care) codes since then) indicating the diagnosis the drug was prescribed for.

The National Population Registry collects demographic information from all residents in Norway since 1960, including information on their date of birth, emigration, immigration and death.

The CACOM database using the Swedish registries

In Sweden, the CACOM database is a compilation of Swedish national health registries and consists of all cases of cancer registered in the Swedish National Cancer Register between 1992-2020, using ICD-10 and ICD-O-3 codes. Since 1958 diagnoses of all incident cancers in Sweden have been mandatorily reported to the Swedish National Cancer Register. The register collects information on the primary site of the tumor, histology, stage, and the date of diagnosis. The case population is approximately 1.5 million individuals. The database also contains a control population randomly sampled from the Total Population Register in Sweden. Each case was matched to 1-3 controls without replacement, on year of birth, gender, county and had to be registered in Sweden at the time of diagnosis. The control population did not have to be cancer-free. Using the unique personal identifier, patients with cancer and population controls were linked to the Swedish Cause of Death Register, and the Prescribed Drug Registry (PDR) in Sweden which contains all prescribed drugs dispensed at pharmacies in Sweden July 1, 2005. The PDR contains information on the dispensed

drug, its classification code according to the World Health Organization Anatomical Therapeutic Chemical System (ATC), dose, amount, and date of dispensing.

Cancer diagnoses are obtained from Swedish Cancer Registry. Since 1958 diagnoses of all incident cancers in Sweden have been mandatorily reported to this register. The register collects information on the primary site of the tumor, histology, stage, and the date of diagnosis.

The SAIL Databank

The SAIL Databank holds anonymised records from numerous sources in Wales. This includes data from national sources such as the cancer registry, register of births and deaths as well as primary and secondary care. The Annual District Death Extract, contains information from the Office for National Statistics (ONS) register of all deaths relating to Welsh residents, including those that died out of Wales since 1996.

The Primary Care GP dataset, captures data from GP practices across Wales, from 2000 to 2020. Data captured includes diagnoses (recorded using Read Code Terminology), symptoms, tests, and referrals for specialist treatment and prescriptions for medications.

The Welsh Cancer Intelligence & Surveillance Unit (WCISU) is the National Cancer Registry of Wales. The WCISU has collected information on all incident cancers in Wales from 1994. Information is available on type (ICD-10 codes), staging, morphology (ICD-O-1-3) and treatment received.

Supplementary Table 1 Definitions of glioma and psychotropic medications

**Malignant neoplasm of brain**	*ICD-10*	C71, C75.1-C75.3,<br>D330, D3311, D332, D430, D431, D432 [where available]
**Gliomas classification**		
**Glioblastoma**	*ICD-0-3*	94403, 94413, 94423
**Diffuse astrocytoma**		94003, 94013
**Oligodendroglioma**[1]		94503, 94513
**Oligoastrocytoma**[1]		93823
**Other**[1]		93803, 93810, 93813, 93831, 93841, 93853, 93913, 93923, 93933, 93941, 93963, 94103, 94113, 94130, 94203, 94211, 94213, 94233, 94243, 94253, 94303, 94311, 94421, 94441, 94453
**All cancer (exclusion criteria)**<br>(excl. non-melanoma skin cancer)	*ICD-10*	C00-97 excl. C44
**Medication classifications**		
	ATC	**Readcodes**
**Psychotropic medication**		
Antidepressants	N06A	d7, d74, d77. d73, d7f, d79, d71, d7c, d7d, d76, d75, d7h, d7a, da4, da9, da5, da3, daC, d8, d83, d81, d84, d85, da, da2, d7b, d7e, da8, d7g, daB, du6, da7, daA, daD, daE

**Antipsychotics**	N05A	d4, d41, d48, d4e, d46, d52, d4b, dhe, d4h, d4a, d4g, d56, d47, d55, ds1, d4i, d42, d44, d49, d45, da1, d51, d43, d4j, d4n, d57, d54, d4c, d4f, d4q, d4y, d4k, d4l, d4r, d58, d4x, d4t, d6, d61, d62 , d4p, d4u, d4v, d4w
**Anxiolytics**	N05B	d2, do1, d24, d2d, d27, d2a, d04, d23, d26, dnc, d22, d2f, d28, d2c
**Hypnotics**	N05C	d31, d11, d05, d15, d18, d1b, d1a, o57, d16, d1d, d1f, d1g, d1h, d1c
**Anti-epileptics**	N03A	dn, dn6, dn7, dna, dn8, dn9, d06, bc6, dni, dn5, dn4, do2, dn3, dnm, dnr, dnu, dnb, dnh, dne, dnl, dnf, dnk, dnj, dno, dnq, dnp, dns, dnt, dnv, dnw, dnx, dA21
**Psychostimulants**	N06B	dc, dc1, dz1, dw2
**Antihistamines**	R06A	c8, dhg, dh4, c73, dh5, dh6, dh9, dhh, dhr, d19, dhf, o59

ATC: Anatomical Therapeutic Chemical; ICD-10: International Classification of Disease 10th Revision; ICD-O-3:

[1] In the new use analysis in Norway and Wales, these categories were collapsed into one category due to low numbers.

Supplementary Table 2 Drugs available in each country during the study period

Drug class	Denmark	Norway	Sweden	Wales
Antiepileptics	Brivaracetam<br>Cannabidiol<br>Carbamazepine<br>Clonazepam<br>Eslicarbazepine<br>Ethosuximide<br>Gabapentin<br>Lacosamide<br>Lamotrigine<br>Levetiracetam<br>Oxcarbazepine<br>Perampanel<br>Phenobarbital<br>Phenytoin<br>Pregabalin<br>Primidone<br>Retigabine<br>Rufinamide<br>Tiagabine<br>Topiramate<br>Valproic acid<br>Vigabatrin<br>Zonisamide	Aminobutyric acid<br>Brivaracetam<br>Cannabidiol<br>Carbamazepine<br>Clonazepam<br>Eslicarbazepine<br>Ethosuximide<br>Felbamate<br>Fosphenytoin<br>Gabapentin<br>Lacosamide<br>Lamotrigine<br>Levetiracetam<br>Mephenytoin<br>Oxcarbazepine<br>Perampanel<br>Phenobarbital<br>Phenytoin<br>Pregabalin<br>Primidone<br>Retigabine<br>Rufinamide<br>Stiripentol<br>Sultiame<br>Tiagabine<br>Topiramate<br>Valproic acid<br>Vigabatrin<br>Zonisamide	Brivaracetam<br>Cannabidiol<br>Carbamazepine<br>Cenobamate<br>Clonazepam<br>Eslicarbazepine<br>Ethosuximide<br>Felbamate<br>Fenfluramine<br>Fosphenytoin<br>Lacosamide<br>Lamotrigine<br>Levetiracetam<br>Oxcarbazepine<br>Perampanel<br>Phenobarbital<br>Phenytoin<br>Primidone<br>Retigabine<br>Rufinamide<br>Stiripentol<br>Sultiame<br>Tiagabine<br>Topiramate<br>Valproic acid<br>Vigabatrin<br>Zonisamide	Brivaracetam<br>Carbamazepine<br>Clonazepam<br>Eslicarbazepine<br>Ethosuximide<br>Fosphenytoin<br>Gabapentin<br>Lacosamide<br>Lamotrigine<br>Levetiracetam<br>Methylphenobarbital<br>Oxcarbazepine<br>Perampanel<br>Phenobarbital<br>Phenytoin<br>Pregabalin<br>Retigabine<br>Rufinamide<br>Stiripentol<br>Tiagabine<br>Topiramate<br>Valproic acid<br>Vigabatrin<br>Zonisamide
Antidepressants	Agomelatine<br>Amitriptyline<br>Bupropion<br>Citalopram<br>Clomipramine<br>Dosulepin<br>Doxepin<br>Duloxetine<br>Escitalopram<br>Fluoxetine<br>Imipramine<br>Lofepramine<br>Maprotiline<br>Mianserin<br>Mirtazapine<br>Moclobemide<br>Nefazodone<br>Nortriptyline<br>Paroxetine<br>Reboxetine<br>sertraline<br>venlafaxine<br>vortioxetine	Agomelatine<br>Amitriptyline<br>Bupropion<br>Citalopram<br>Clomipramine<br>Desipramine<br>Dibenzepin<br>Doxepin<br>Duloxetine<br>Escitalopram<br>Esketamine<br>Fluoxetine<br>Fluvoxamine<br>Hyperici herba<br>Imipramine<br>Isocarboxazid<br>Lofepramine<br>Maprotiline<br>Mianserin<br>Mirtazapine<br>Moclobemide<br>Nefazodone<br>Nortriptyline<br>Opipramol<br>Oxitriptan	Agomelatine<br>Amitriptyline<br>Bupropion<br>Citalopram<br>Clomipramine<br>Desipramine<br>Desvenlafaxine<br>Dosulepin<br>Doxepin<br>Duloxetine<br>Escitalopram<br>Esketamine<br>Fluoxetine<br>Fluoxetine<br>Fluvoxamine<br>Imipramine<br>Isocarboxazid<br>Lofepramine<br>Maprotiline<br>Mianserin<br>Milnacipran	Agomelatine<br>Amitriptyline<br>Amoxapine<br>Bupropion<br>Citalopram<br>Clomipramine<br>Desipramine<br>Dosulepin<br>Doxepin<br>Duloxetine<br>Escitalopram<br>Fluoxetine<br>Fluvoxamine<br>Imipramine<br>Isocarboxazid<br>Lofepramine<br>Maprotiline<br>Mianserin<br>Mirtazapine<br>Moclobemide<br>Nefazodone

**Drug class**	**Denmark**	**Norway**	**Sweden**	**Wales**
		Paroxetine<br>Phenelzine<br>Protriptyline<br>Reboxetine<br>Sertraline<br>Tianeptine<br>Tranylcypromine<br>Trazodone<br>Trimipramine<br>Tryptophan<br>Venlafaxine<br>Viloxazine<br>Vortioxetine	Mirtazapine<br>Moclobemide<br>Nefazodone<br>Nortriptyline<br>Opipramol<br>Oxitriptan<br>Paroxetine<br>Phenelzine<br>Protriptyline<br>Reboxetine<br>Sertraline<br>Tianeptine<br>Tranylcypromine<br>Trazodone<br>Trimipramine<br>Tryptophan<br>Venlafaxine<br>Vilazodone<br>Vortioxetine	Nortriptyline<br>Paroxetine<br>Phenelzine<br>Protriptyline<br>Reboxetine<br>Sertraline<br>Tranylcypromine<br>Trazodone<br>Trimipramine<br>Tryptophan<br>Venlafaxine<br>Viloxazine<br>Vortioxetine
Hypnotics & Sedatives	Brotizolam<br>Clomethiazole<br>Estazolam<br>Flunitrazepam<br>Lormetazepam<br>Melatonin<br>Midazolam<br>Nitrazepam<br>Triazolam<br>Zaleplon<br>Zolpidem<br>Zopiclone	Barbital<br>Barbituates in combination<br>Bromides<br>Chloral hydrate<br>Clomethiazole<br>Dexmedetomidine<br>Estazolam<br>Flunitrazepam<br>Flurazepam<br>Melatonin<br>Midazolam<br>Nitrazepam<br>Pentobarbital<br>Propiomazine<br>Scopolamine<br>Secobarbital<br>Triazolam<br>Valerianae radix<br>Zaleplon<br>Zolpidem<br>Zopiclone	Chloral hydrate<br>Clomethiazole<br>Daridorexant<br>Flunitrazepam<br>Lemborexant<br>Melatonin<br>Midazolam<br>Nitrazepam<br>Propiomazine<br>Scopolamine<br>Suvorexant<br>Triazolam<br>Valerianae radix<br>Zaleplon<br>Zolpidem<br>Zopiclone	Amobarbital<br>Butobarbital<br>Chloral hydrate<br>Clomethiazole<br>Combinations of barbiturates<br>Cyclobarbital<br>Flunitrazepam<br>Flurazepam<br>Loprazolam<br>Lormetazepam<br>Melatonin<br>Midazolam<br>Nitrazepam<br>Paraldehyde<br>Secobarbital<br>Temazepam<br>Triazolam<br>Triclofos<br>Zaleplon<br>Zolpidem<br>Zopiclone
Anxiolytics	Alprazolam<br>Bromazepam<br>Buspirone<br>Chlordiazepoxide<br>Clobazam<br>Diazepam<br>Hydroxyzine<br>Lorazepam<br>Oxazepam	Alprazolam<br>Bromazepam<br>Buspirone<br>Chlordiazepoxide<br>Clobazam<br>Diazepam<br>Hydroxyzine<br>Lorazepam<br>Meprobamate	Alprazolam<br>Bromazepam<br>Buspirone<br>Chlordiazepoxide<br>Clobazam<br>Diazepam<br>Hydroxyzine<br>Lorazepam<br>Meprobamate	Alprazolam<br>Bromazepam<br>Buspirone<br>Chlordiazepoxide<br>Clobazam<br>Diazepam<br>Hydroxyzine<br>Lorazepam<br>Medazepam

Drug class	Denmark	Norway	Sweden	Wales
	Potassium clorazepate	Oxazepam<br>Potassium clorazepate	Oxazepam<br>Potassium clorazepate	Meprobamate<br>Oxazepam<br>Potassium clorazepate
Antipsychotics	Acepromazine<br>Amisulpride<br>Aripiprazole<br>Asenapine<br>Chlorpromazine<br>Chlorprothixene<br>Clozapine<br>Droperidol<br>Flupentixol<br>Fluphenazine<br>Haloperidol<br>Levomepromazine<br>Lithium<br>Melperone<br>Olanzapine<br>Paliperidone<br>Penfluridol<br>Periciazine<br>Perphenazine<br>Pimozide<br>Pipamperone<br>Prochlorperazine<br>Quetiapine<br>Risperidone<br>Sertindole<br>Sulpiride<br>Thioridazine<br>Ziprasidone<br>Zuclopenthixol	Amisulpride<br>Aripiprazole<br>Asenapine<br>Brexpiprazole<br>Cariprazine<br>Chlorpromazine<br>Chlorprothixene<br>Clozapine<br>Dixyrazine<br>Droperidol<br>Flupentixol<br>Fluphenazine<br>Fluspirilene<br>Haloperidol<br>Levomepromazine<br>Lithium<br>Loxapine<br>Lurasidone<br>Melperone<br>Olanzapine<br>Paliperidone<br>Penfluridol<br>Perazine<br>Periciazine<br>Perphenazine<br>Pimozide<br>Pipotiazine<br>Prochlorperazine<br>Prothipendyl<br>Quetiapine<br>Risperidone<br>Sertindole<br>Sulpiride<br>Thioproperazine<br>Thioridazine<br>Tiapride<br>Trifluoperazine<br>Ziprasidone<br>Zuclopenthixol	Acepromazine<br>Amisulpride<br>Aripiprazole<br>Brexpiprazole<br>Cariprazine<br>Chlorpromazine<br>Chlorprothixene<br>Clozapine<br>Cyamemazine<br>Dixyrazine<br>Droperidol<br>Flupentixol<br>Fluphenazine<br>Fluspirilene<br>Haloperidol<br>Levomepromazine<br>Lithium<br>Loxapine<br>Lumateperone<br>Lurasidone<br>Melperone<br>Olanzapine<br>Paliperidone<br>Penfluridol<br>Perazine<br>Periciazine<br>Perphenazine<br>Pimavanserin<br>Pimozide<br>Pipamperone<br>Prochlorperazine<br>Quetiapine<br>Risperidone<br>Sertindole<br>Sulpiride<br>Thioridazine<br>Tiapride<br>Trifluoperazine<br>Ziprasidone<br>Zuclopenthixol	Amisulpride<br>Aripiprazole<br>Asenapine<br>Benperidol<br>Chlorpromazine<br>Clozapine<br>Droperidol<br>Flupentixol<br>Fluphenazine<br>Fluspirilene<br>Haloperidol<br>Levomepromazine<br>Lithium<br>Loxapine<br>Lurasidone<br>Olanzapine<br>Oxypertine<br>Paliperidone<br>Periciazine<br>Perphenazine<br>Pimozide<br>Pipotiazine<br>Prochlorperazine<br>Promazine<br>Quetiapine<br>Risperidone<br>Sertindole<br>Sulpiride<br>Thioridazine<br>Trifluoperazine<br>Trifluperidol<br>Zotepine<br>Zuclopenthixol
Psychostimulant	Amfetamine<br>Atomoxetine<br>Dexamfetamine<br>Lisdexamfetamine<br>Methylphenidate<br>Modafinil	Adrafinil<br>Amfetamine<br>Atomoxetine<br>Caffeine<br>Dexamfetamine<br>Idebenone<br>Lisdexamfetamine<br>Methylphenidate<br>Modafinil<br>Pemoline<br>Piracetam<br>Propentofylline	Amfetamine<br>Armodafinil<br>Atomoxetine<br>Caffeine<br>Dexamfetamine<br>Dexmethylphenidate<br>Idebenone<br>Lisdexamfetamine<br>Methylphenidate<br>Modafinil<br>Piracetam<br>Solriamfetol	Atomoxetine<br>Dexamfetamine<br>Lisdexamfetamine<br>Methylphenidate<br>Modafinil
Antihistamines	Acrivastine	Acrivastine	Acrivastine	

Drug class	Denmark	Norway	Sweden	Wales
	Astemizole<br>Bilastine<br>Cetirizine<br>Chlorcyclizine<br>Clemastine<br>Cyclizine<br>Cyproheptadine<br>Desloratadine<br>Dexchlorpheniramine<br>Diphenhydramine<br>Ebastine<br>Fexofenadine<br>Levocetirizine<br>Loratadine<br>Meclozine<br>Methdilazine<br>Mizolastine<br>Promethazine<br>Terfenadine	Alimemazine<br>Astemizole<br>Bilastine<br>Cetirizine<br>Chlorcyclizine<br>Clemastine<br>Cyclizine<br>Cyproheptadine<br>Desloratadine<br>Dexchlorpheniramine<br>Dimetindene<br>Diphenhydramine<br>Diphenhydramine, combinations<br>Doxylamine<br>Ebastine<br>Fexofenadine<br>Isothipendyl<br>Ketotifen<br>Levocetirizine<br>Loratadine<br>Meclozine<br>Meclozine, combinations<br>Phenindamine<br>Promethazine<br>Promethazine, combinations<br>Rupatadine<br>Terfenadine<br>Thiethylperazine	Alimemazine<br>Bilastine<br>Cetirizine<br>Clemastine<br>Cyclizine<br>Cyproheptadine<br>Desloratadine<br>Dexchlorpheniramine<br>Dimenhydrinate<br>Diphenhydramine<br>Doxylamine, combinations<br>Ebastine<br>Fexofenadine<br>Ketotifen<br>Levocetirizine<br>Loratadine<br>Meclozine<br>Mizolastine<br>Oxatomide<br>Promethazine<br>Promethazine, combinations<br>Rupatadine<br>Thiethylperazine	Acrivastine<br>Alimemazine<br>Astemizole<br>Bilastine<br>Brompheniramine<br>Cetirizine<br>Chlorcyclizine<br>Chlorphenamine<br>Clemastine<br>Cyclizine<br>Cyproheptadine<br>Desloratadine<br>Dexchlorpheniramine<br>Diphenhydramine<br>Ebastine<br>Fexofenadine<br>Ketotifen<br>Levocetirizine<br>Loratadine<br>Meclozine<br>Methdilazine<br>Mizolastine<br>Promethazine<br>Rupatadine<br>Terfenadine

Supplementary Figure 1 Flow chart for inclusion, by study site

	Denmark	Norway	Sweden	Wales
Incident glioma >18 years (≥21 years in Wales)	2001-2018<br>n=11,157	2004-2021<br>n=11,998	2006-2020<br>n=16,276	2003-2016<br>n=4,702
	↓	↓	↓	↓
Exclude post-mortem diagnosis	n=11,102	n=11,941	n=16,197	n=4,577
	↓	↓	↓	↓
Exclude no histology	n=6,616	n=6,052	n=8,126	n=3,445
	↓	↓	↓	↓
Exclude those with coverage at time of diagnosis	n/a	n=6,050	n=8,032	n=2,739
	↓	↓	↓	↓
Exclude previous cancers	n=6,080	n= 5,458	n=6,883	n=2,579
	↓	↓	↓	↓
Exclude <2 years baseline availability*	n=5,586	n=4,844	n=6,797	n=2,042
	↓	↓	↓	↓
Restricting study period to ensure sufficient look-back & follow up for prescription use	n=4,942	n=4,814	n=5,361	n=1,567
	↓	↓	↓	↓

Excluding glioma cases with no matched controls	n/a	n/a	n/a	n=1,494
	⬇	⬇	⬇	⬇
Final study population	2001-2016<br>4,942 glioma cases<br>49,420 matched controls	2006-2019<br>4,210 glioma cases<br>38,687 matched controls	2008-2018<br>5,361 glioma cases<br>40,315 matched controls	2005-2016<br>1,494 glioma cases<br>12,879 matched controls

* <3 years in Wales

Supplementary Figure 2 Rates of new psychotropic prescriptions in 1-month intervals before and after the month of glioma diagnosis among glioma patients and age and sex matched comparison cohort by histological subtype and study site.

A) Glioblastoma

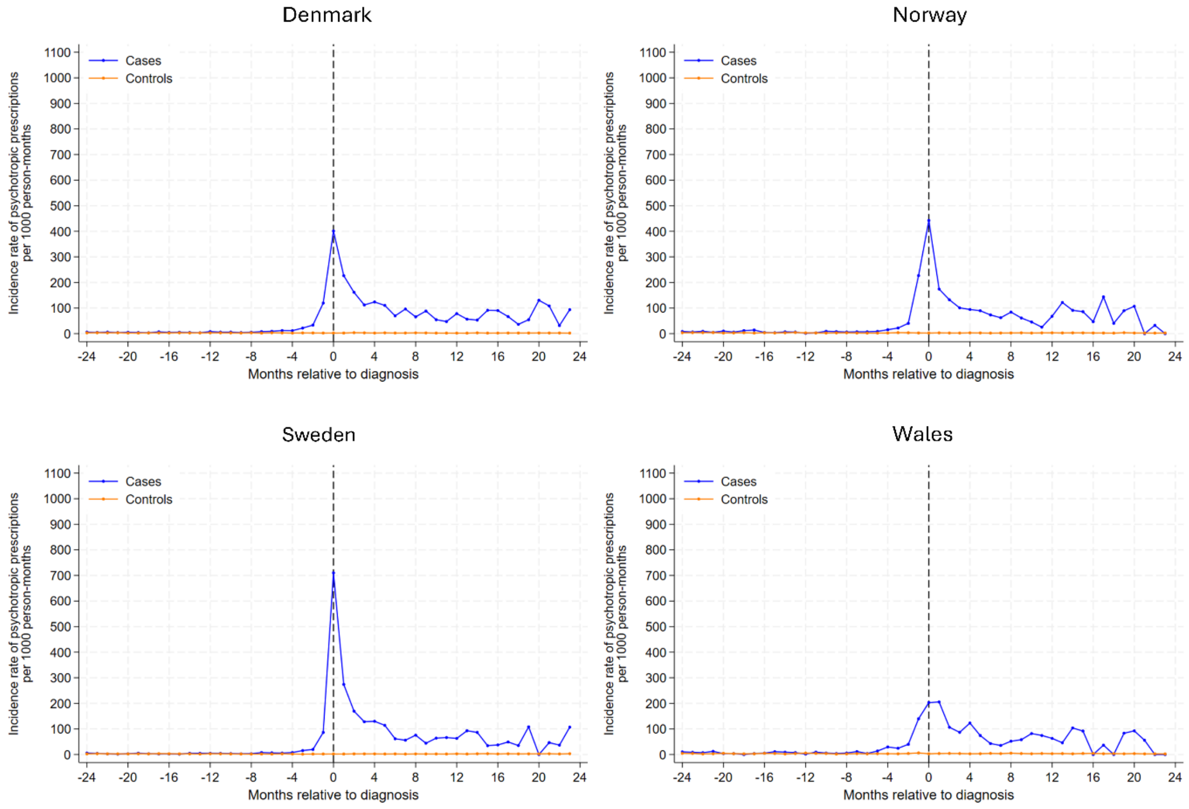

Supplementary Figure 2 (continued)

B) Diffuse astrocytoma

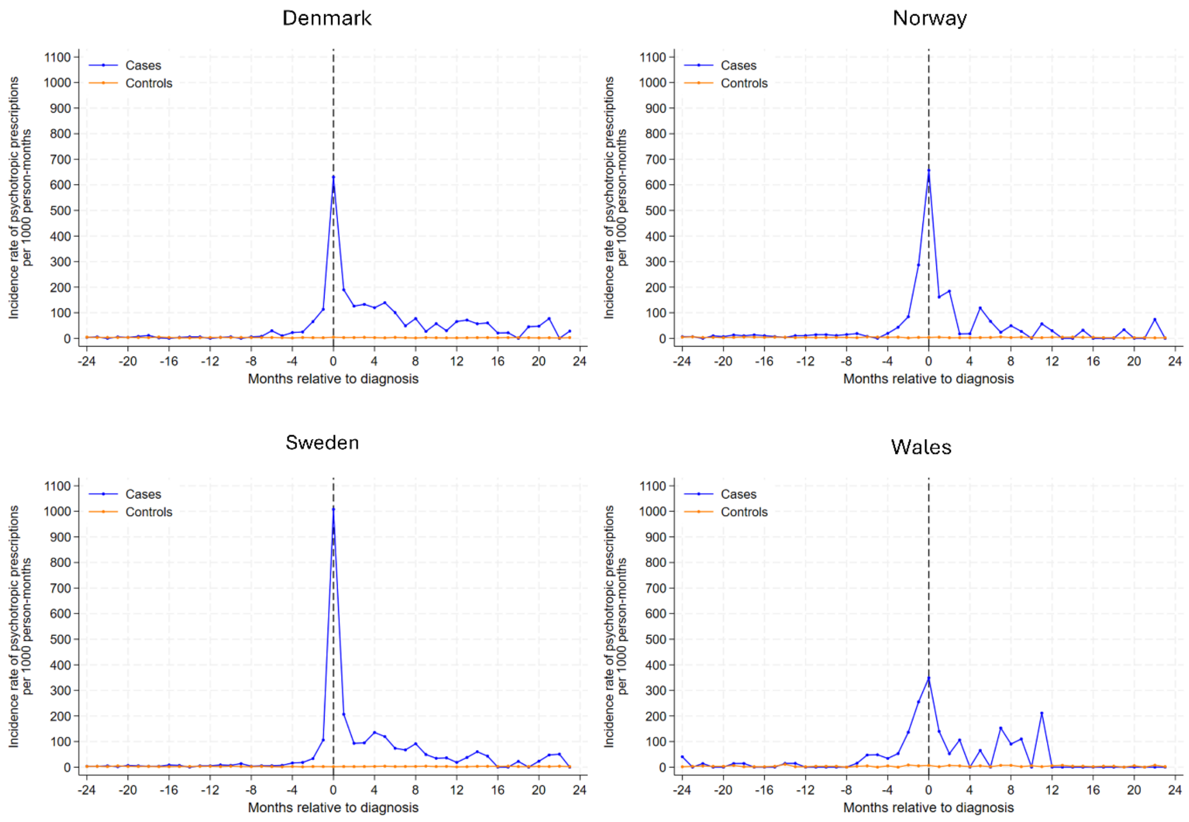

C) Oligodendroglioma

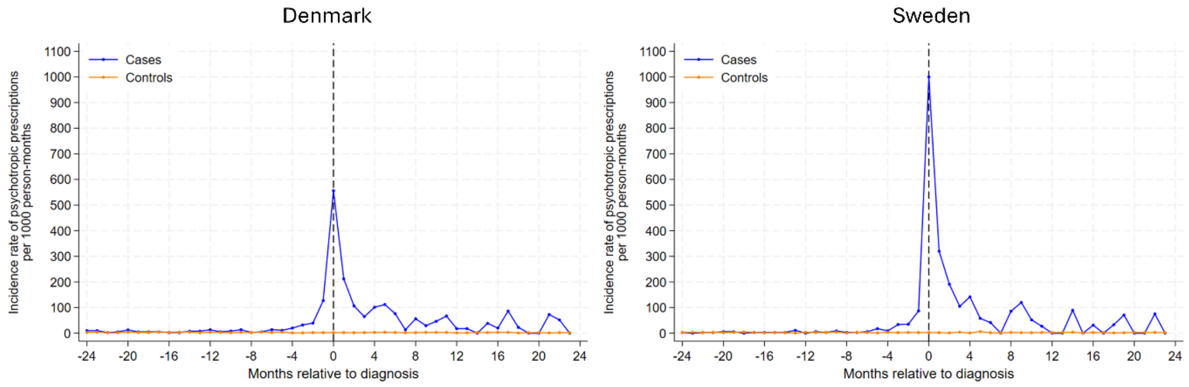

D) Oligoastrocytoma

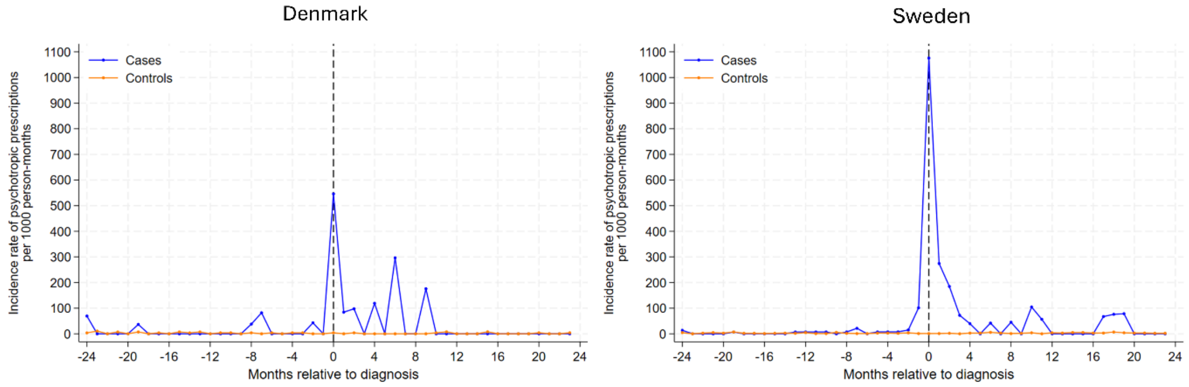

Supplementary Figure 2 (continued)

E) Other glioma

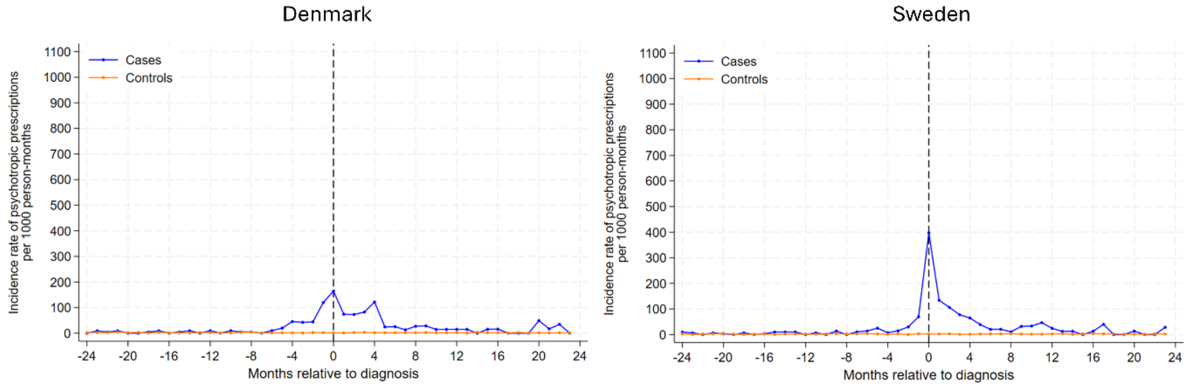

F) Oligodendroglioma, oligoastrocytoma and other glioma (Norway and Wales only)

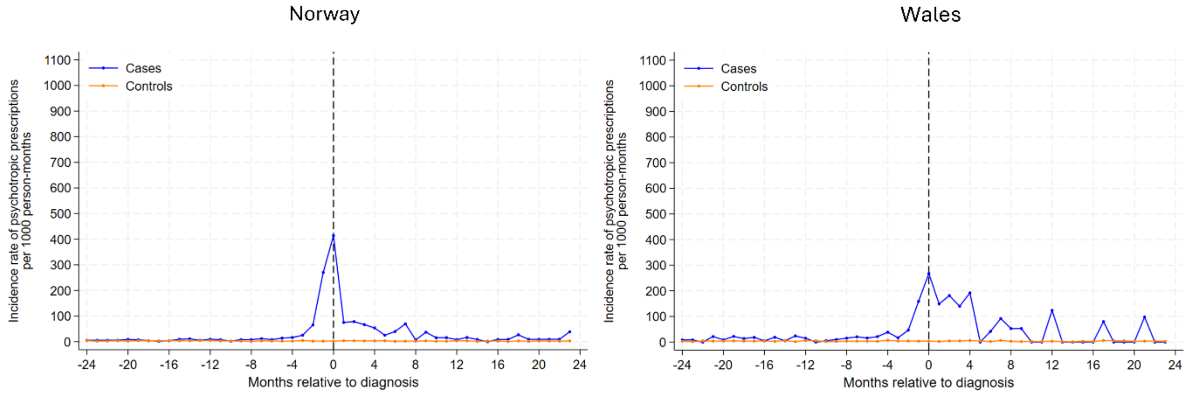

Supplementary Figure 3 Rates of all prescriptions for psychotropic medication in 1-month intervals before and after month of glioma diagnosis among glioma patients or among age and sex matched comparison cohort by study site.

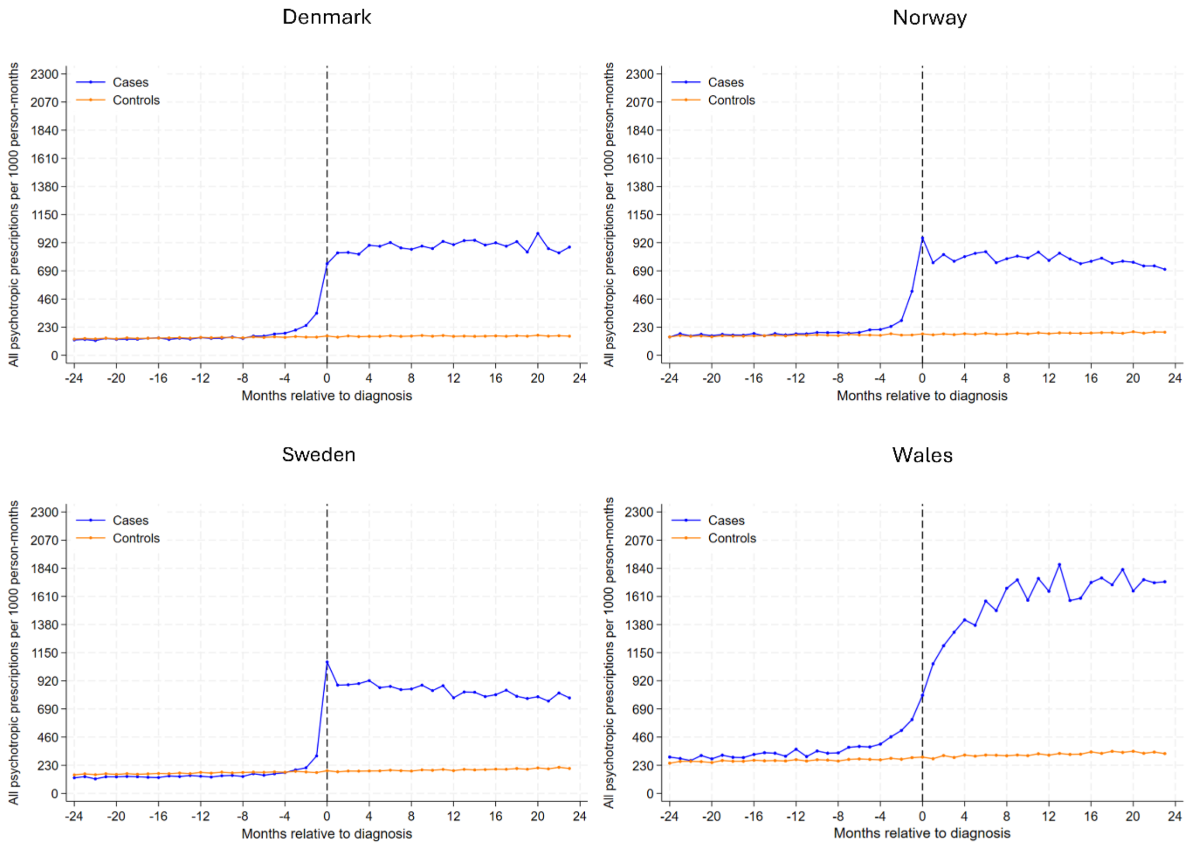

Supplementary Figure 4 Rates of all prescriptions for psychotropic medication in 1-month intervals before and after month of glioma diagnosis among glioma patients or among age and sex matched comparison cohort by histological subtype and study site.

A) Glioblastoma

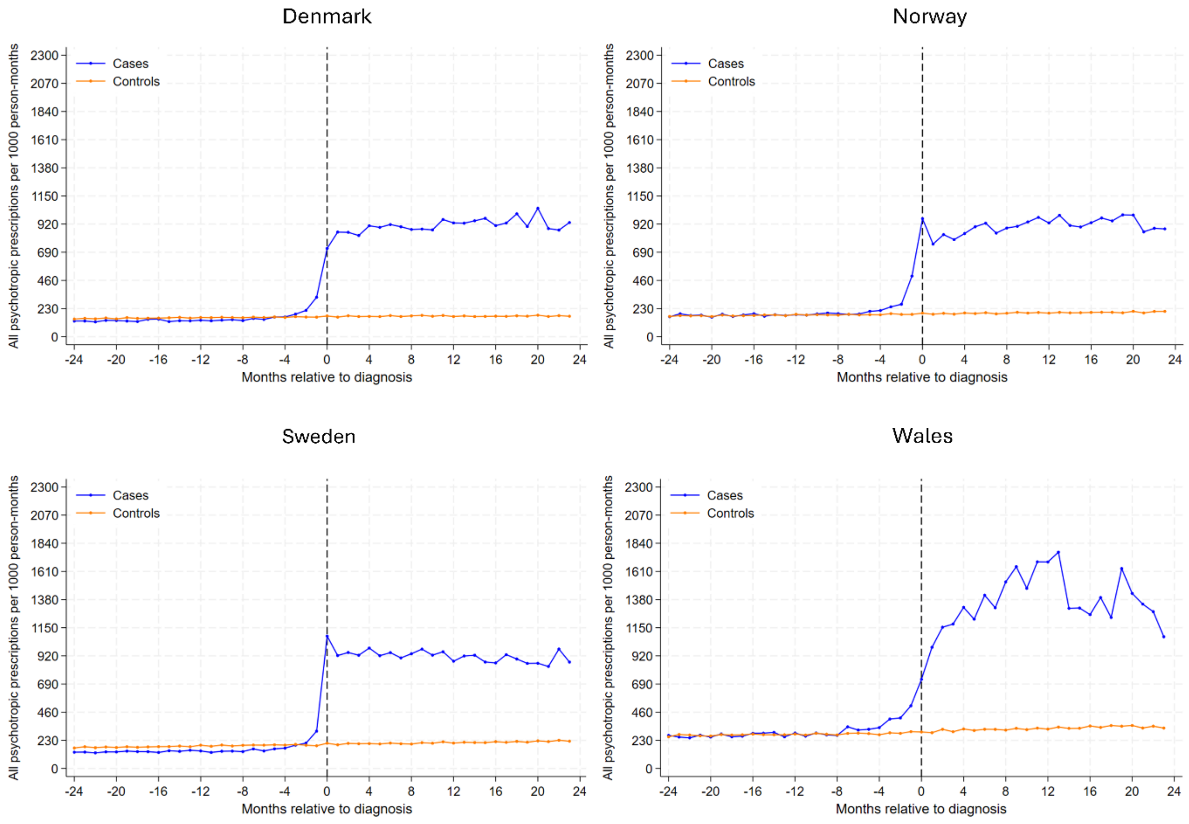

Supplementary Figure 4 (continued)

B) Diffuse astrocytoma

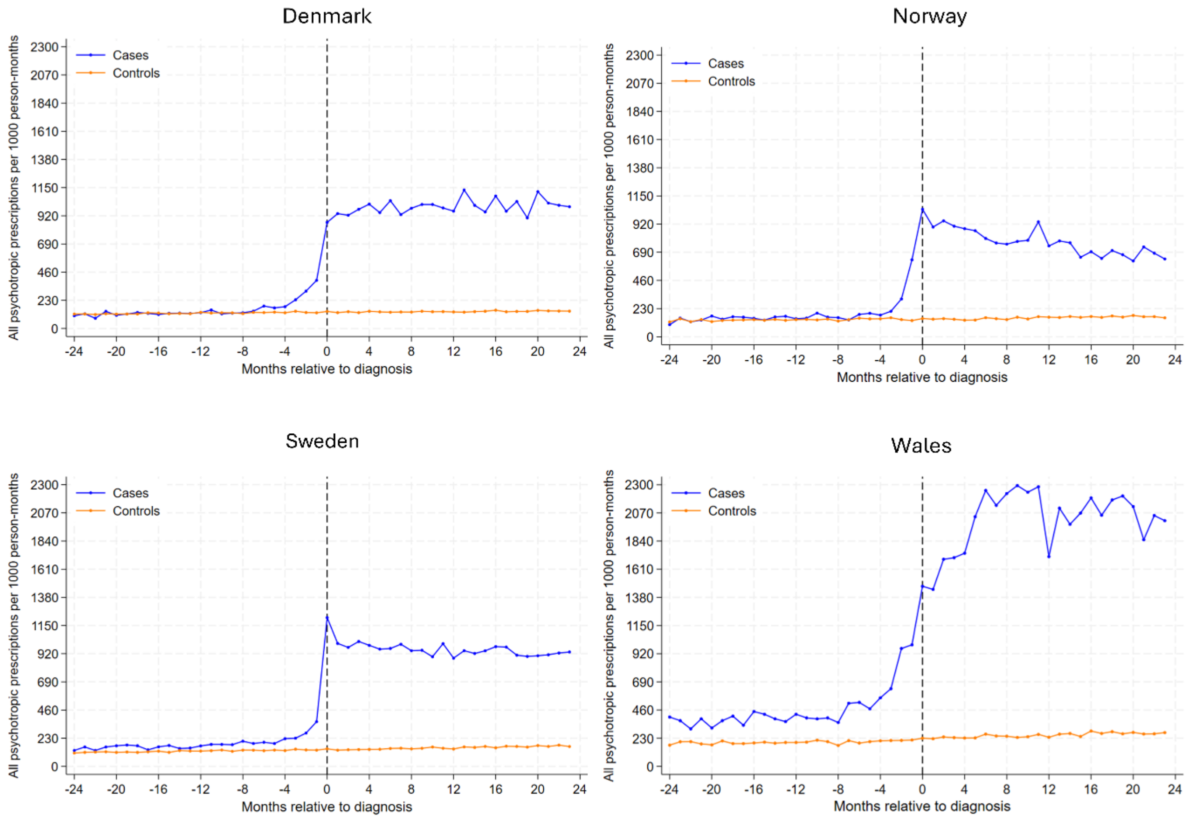

C) Oligodendroglioma

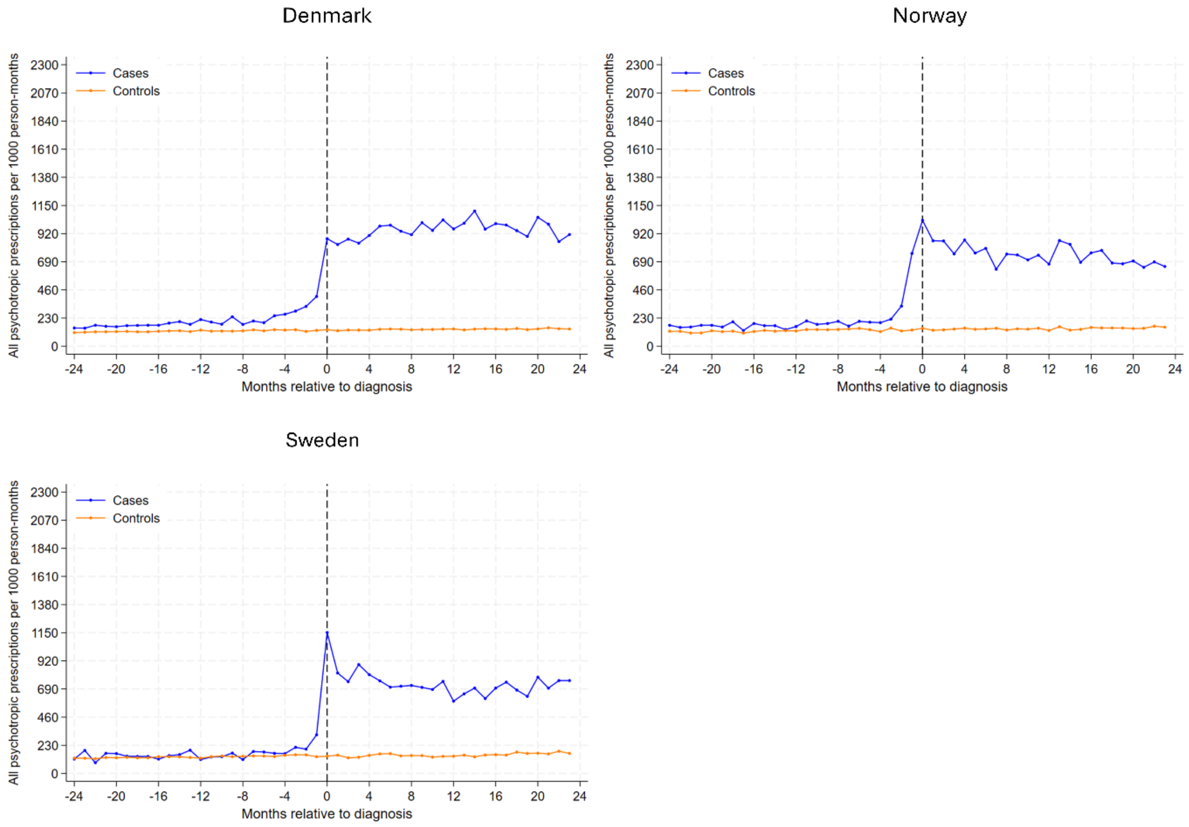

Supplementary Figure 4 (continued)

D) Oligoastrocytoma

Denmark

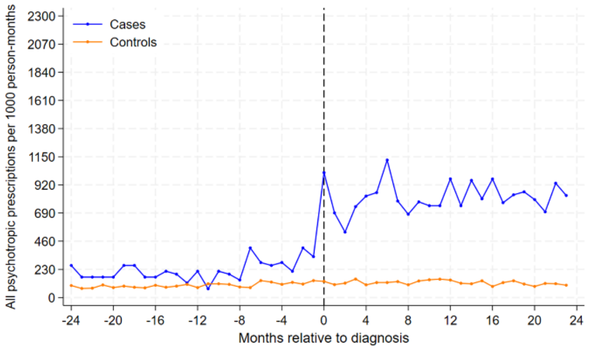

Norway

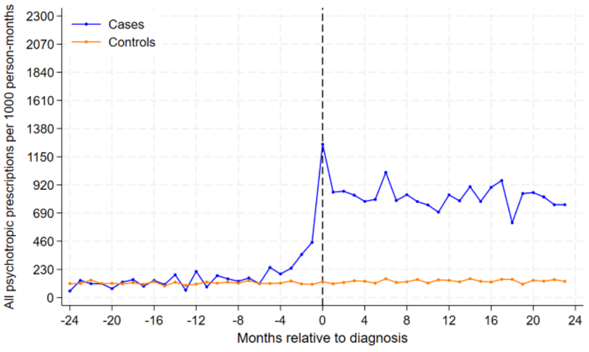

Sweden

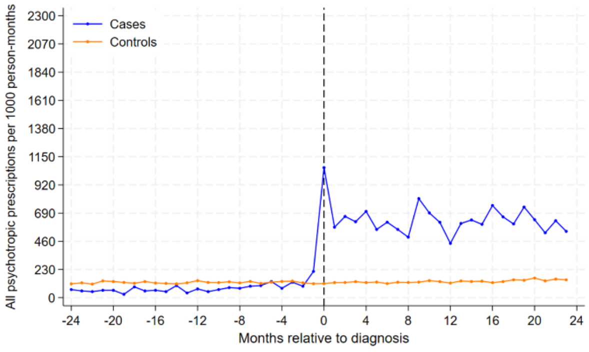

E) Other glioma

Denmark

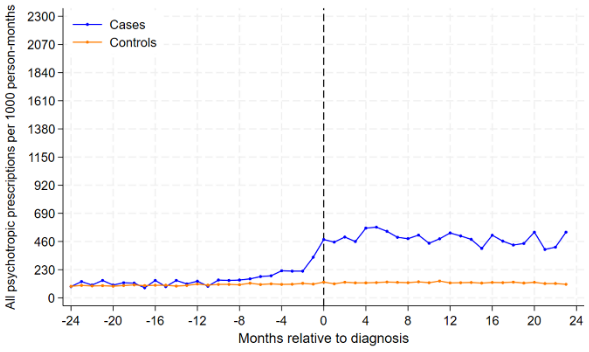

Norway

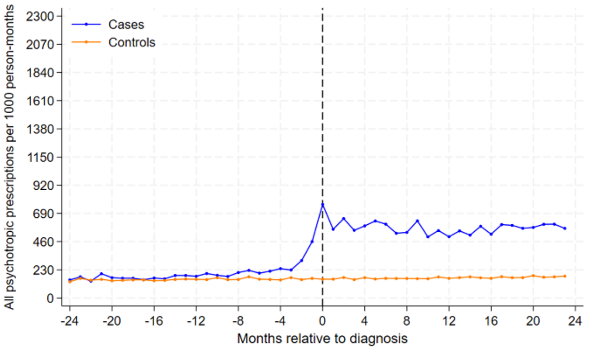

Sweden

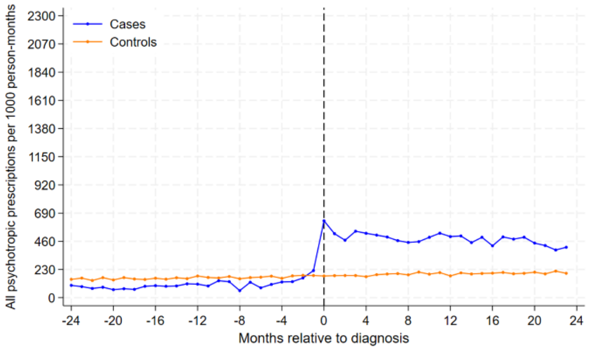

Supplementary Figure 4 (continued)

F) Oligodendroglioma, oligoastrocytoma and other glioma (Wales only)

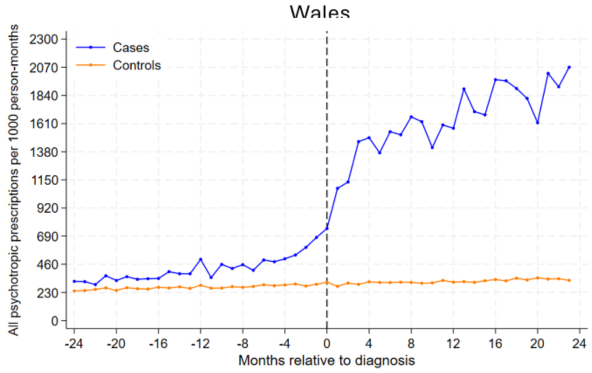

Supplementary Figure 5 Rates of all prescriptions for psychotropic medication in 1-month intervals before and after month of glioma diagnosis among glioma patients or among age and sex matched comparison cohort by individual drug class and study site. Note different y axis.

A) Antiepileptics (N03A)

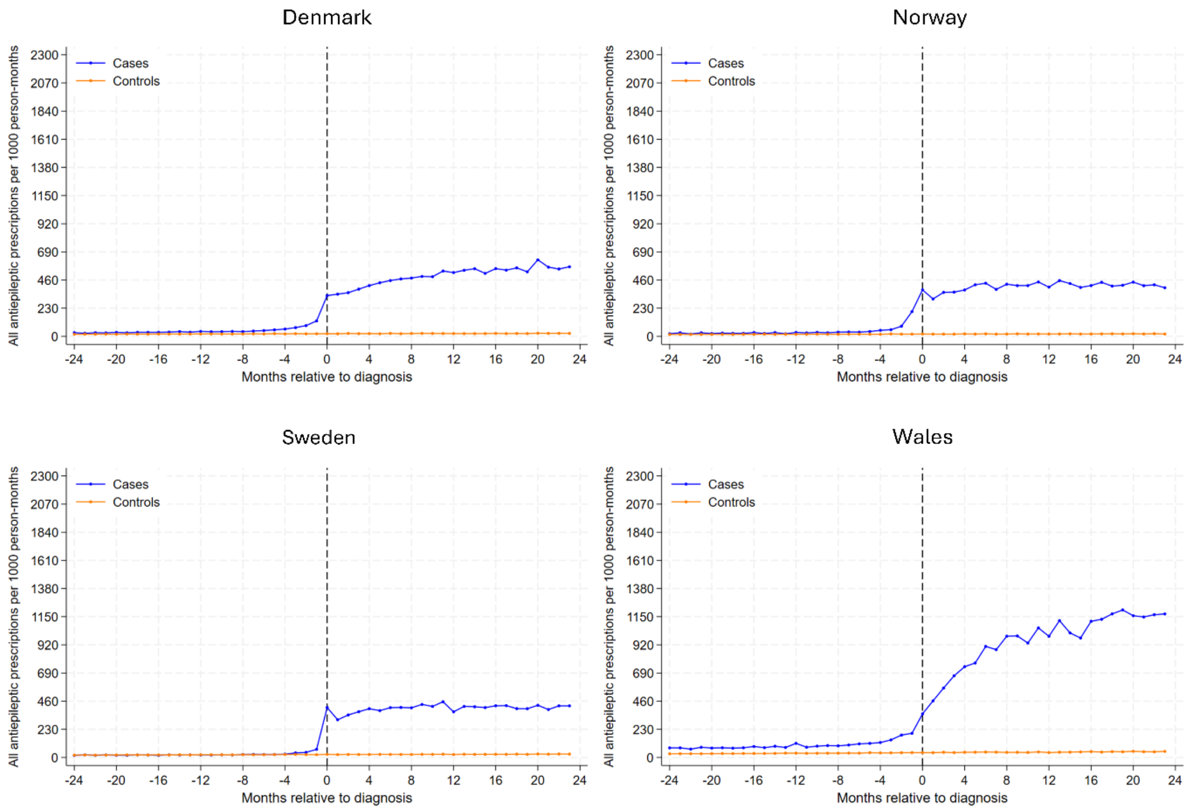

Supplementary Figure 5 (continued)

B) Hypnotics (N05C)

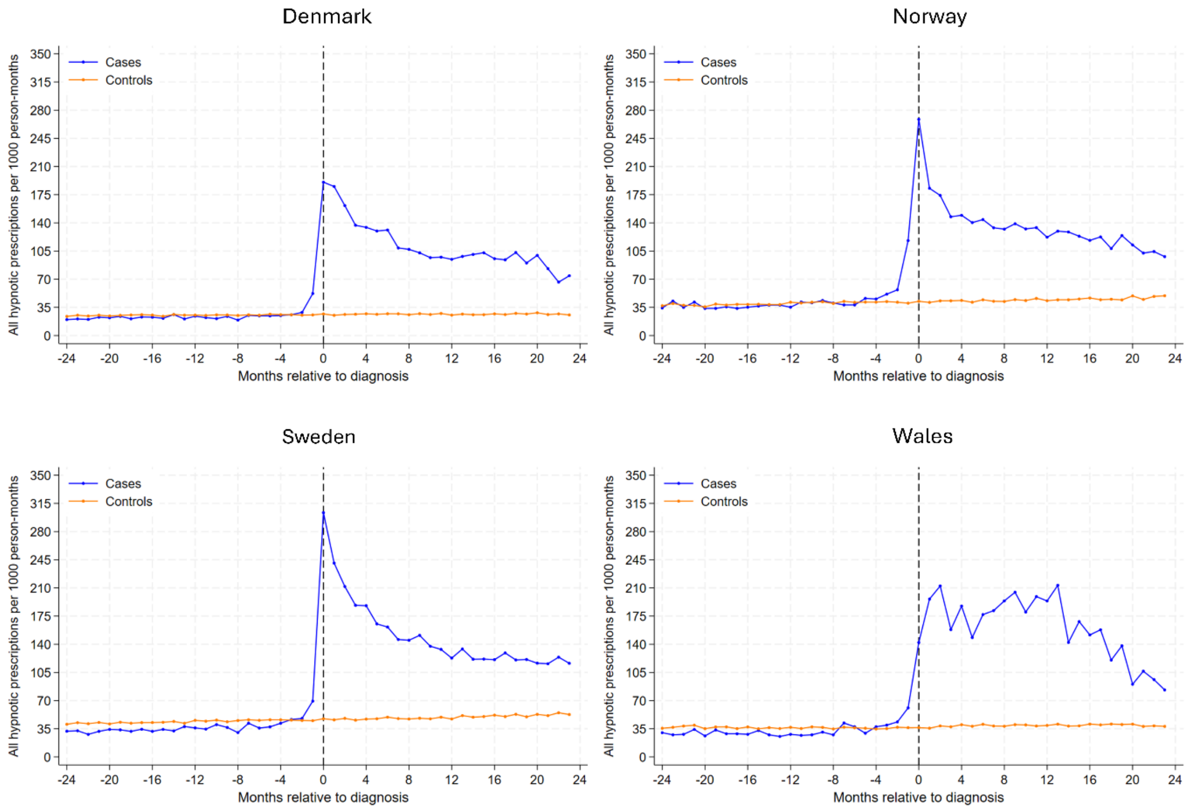

C) Anxiolytics (N05B)

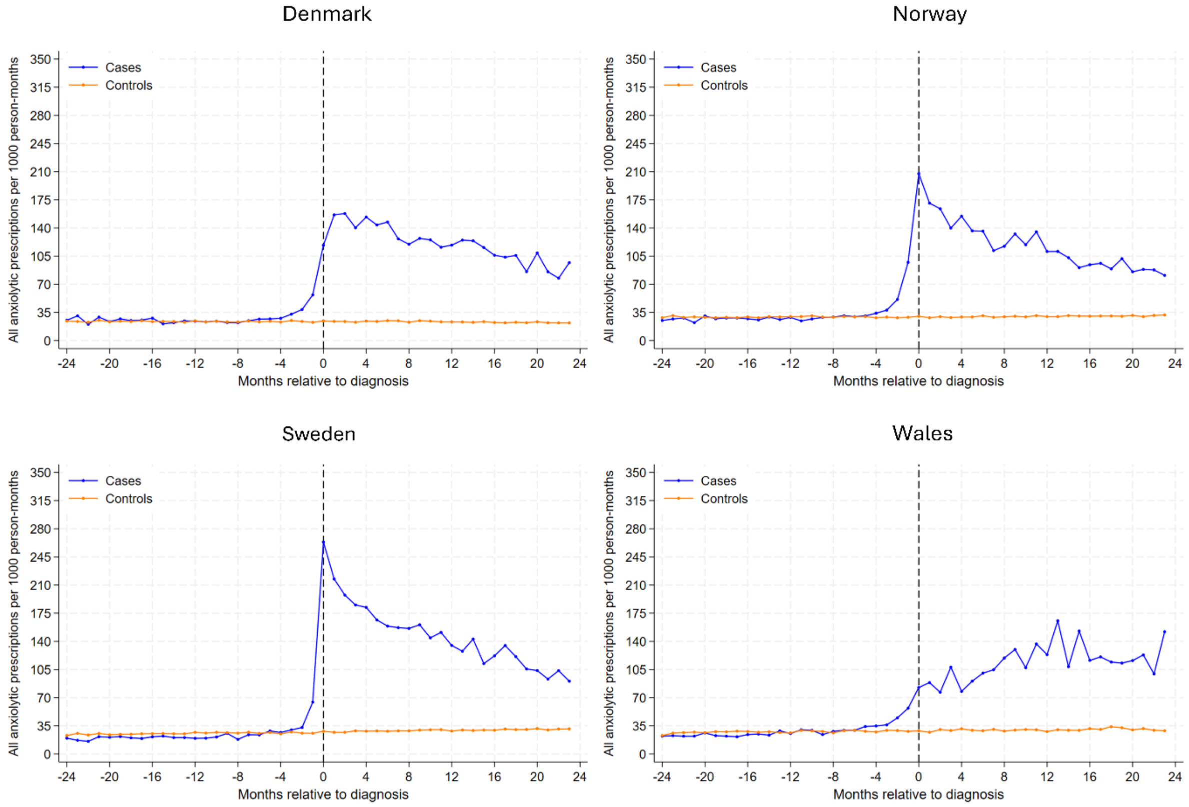

Supplementary Figure 5 (continued)

D) Antidepressants (N06A)

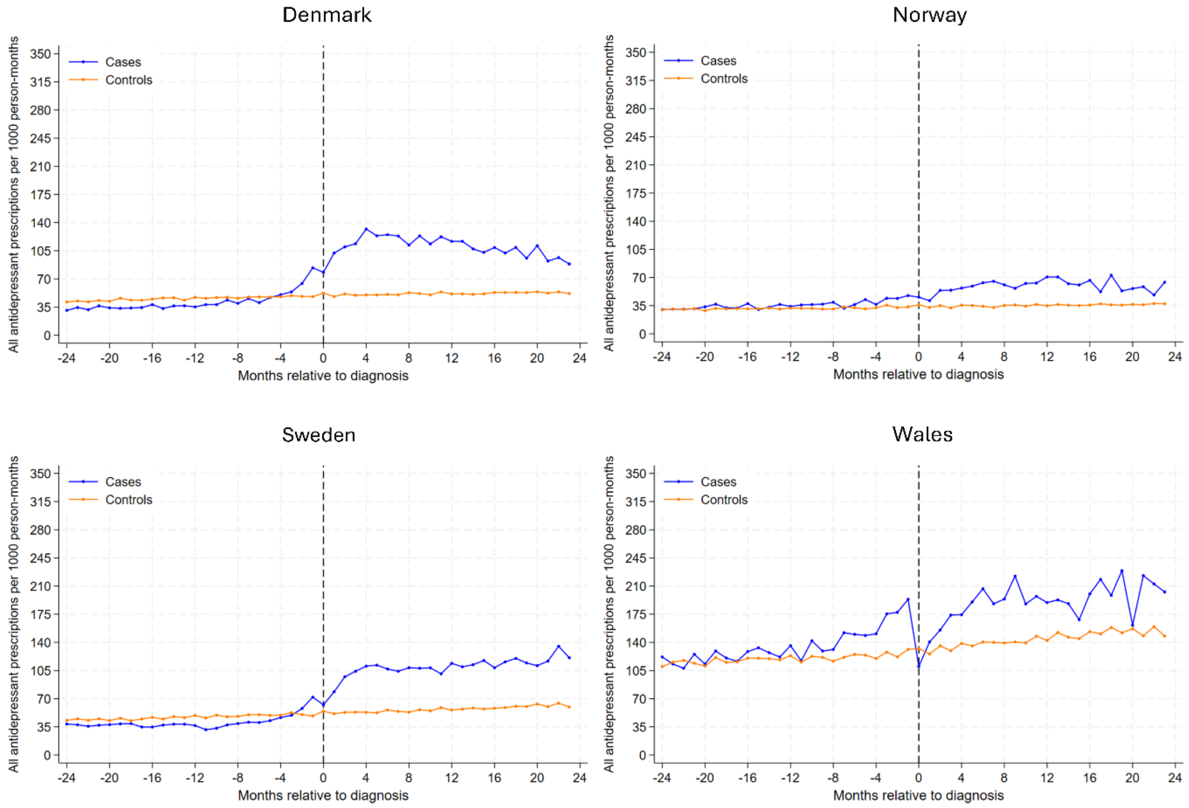

E) Antipsychotics (N05A)

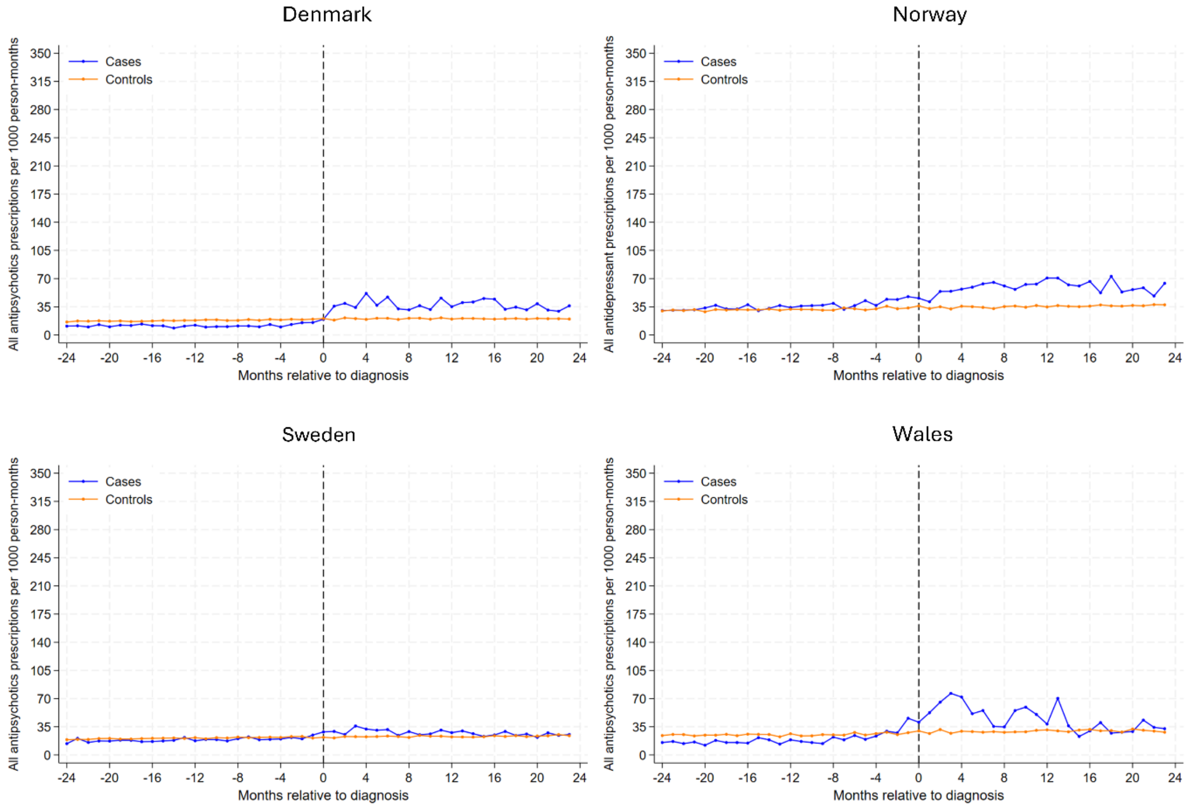

Supplementary Figure 5 (continued)

F) Antihistamines (R06A)

Denmark

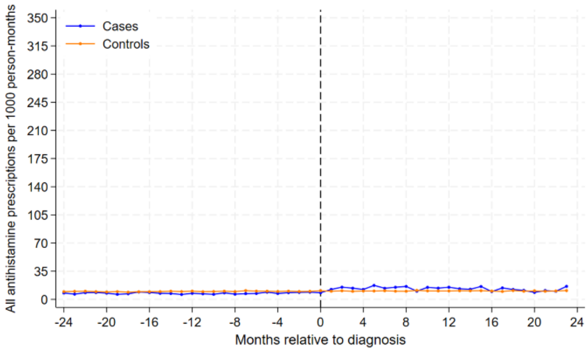

Norway

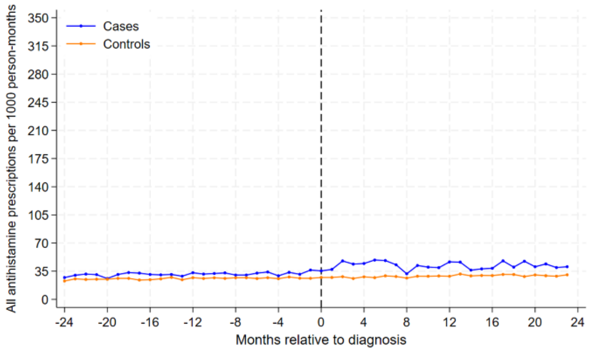

Sweden

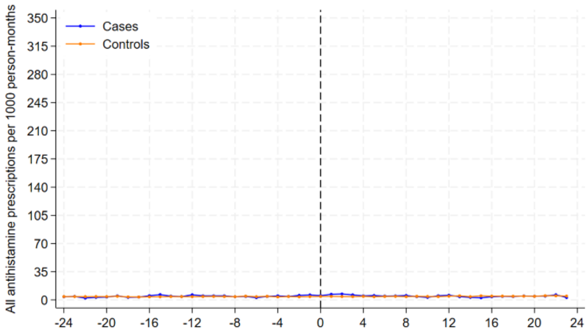

Wales

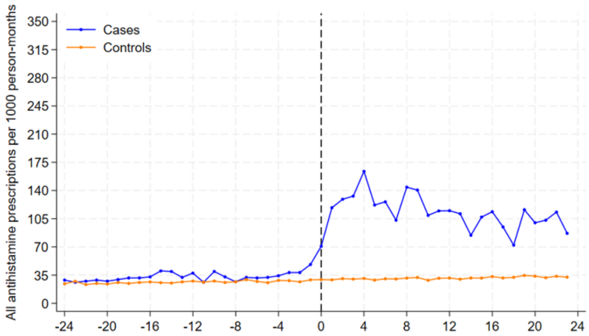

G) Psychostimulants (N06B)

Denmark

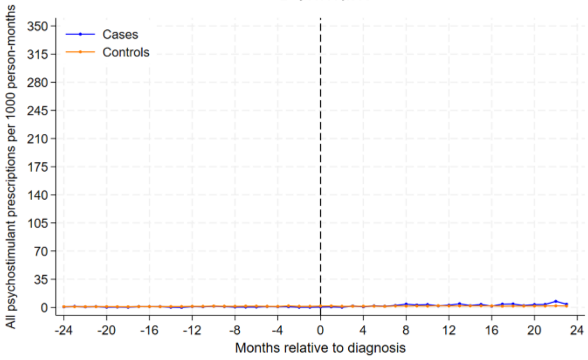

Norway

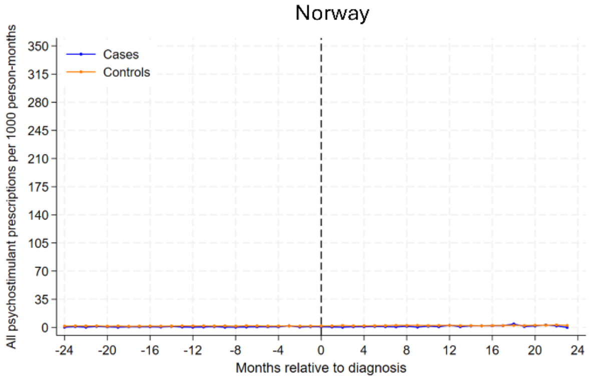

Sweden

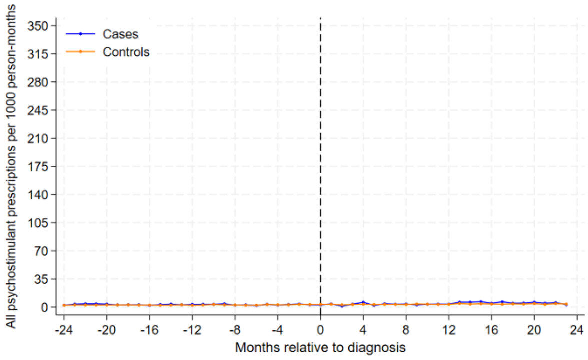

Wales

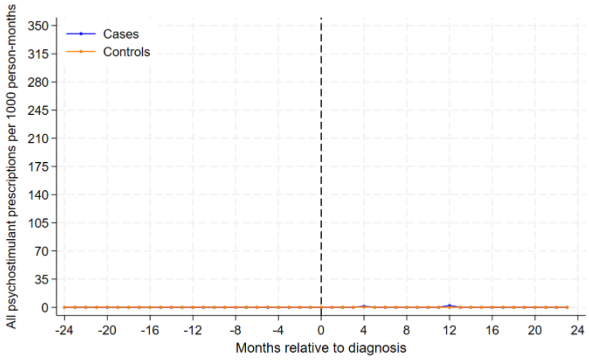

Supplementary Figure 6 Rates of DDDs for psychotropic medication in 1-month intervals before and after month of glioma diagnosis among glioma patients or among age and sex matched comparison cohort by individual drug class and study site. Note different y axis.

A) Antiepileptics (N03A)

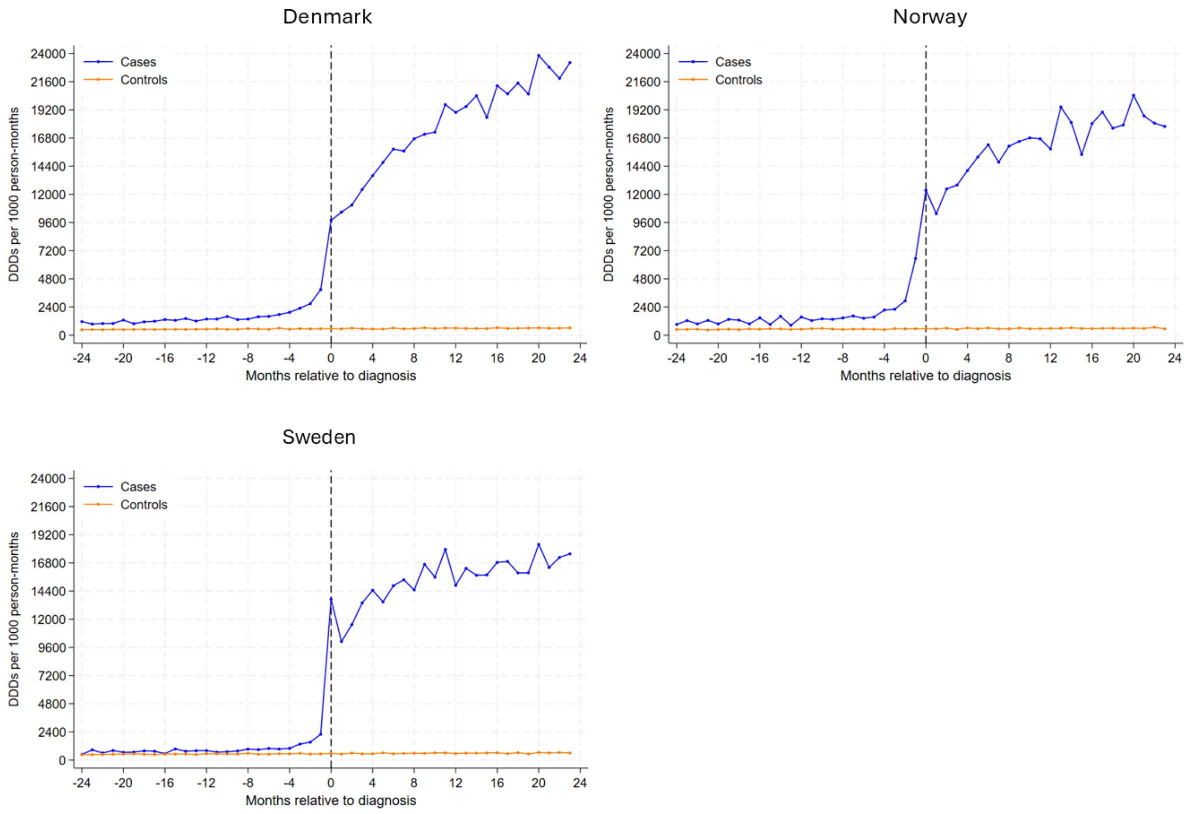

Supplementary Figure 6 (continued)

B) Hypnotics (N05C)

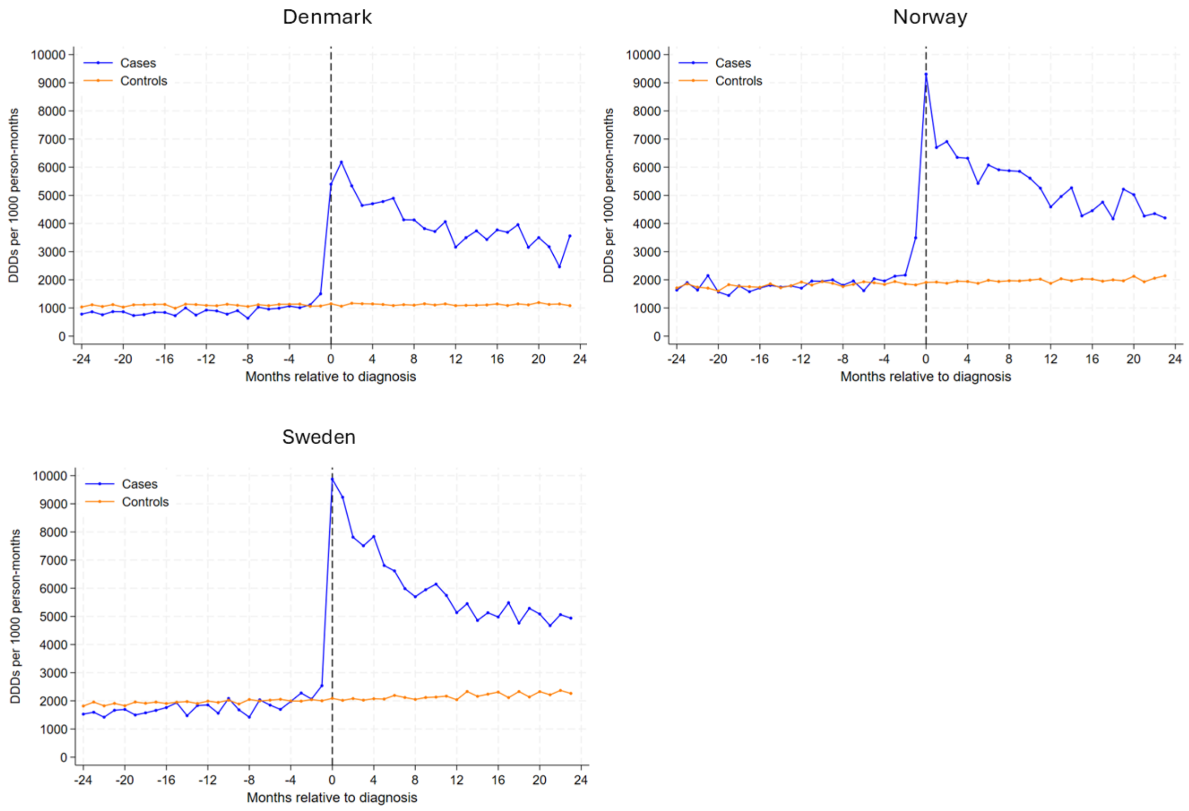

C) Anxiolytics (N05B)

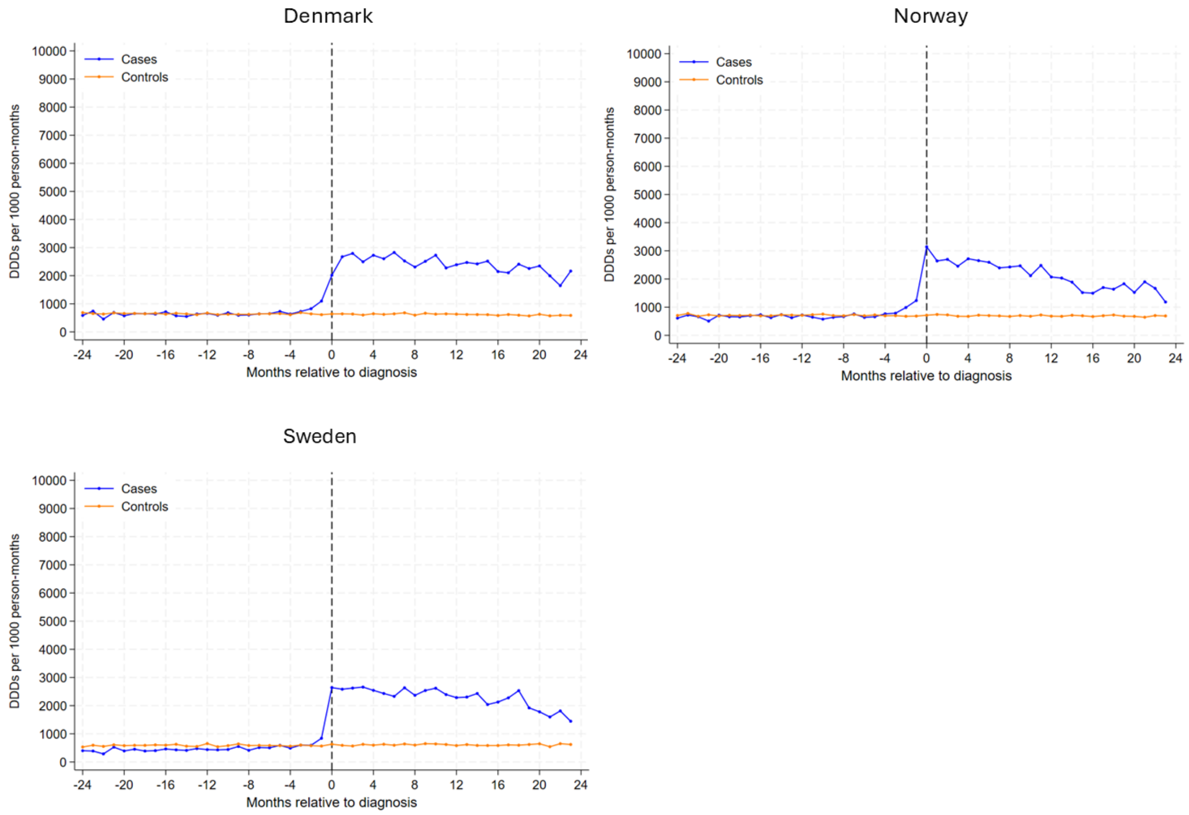

Supplementary Figure 6 (continued)

D) Antidepressants (N06A)

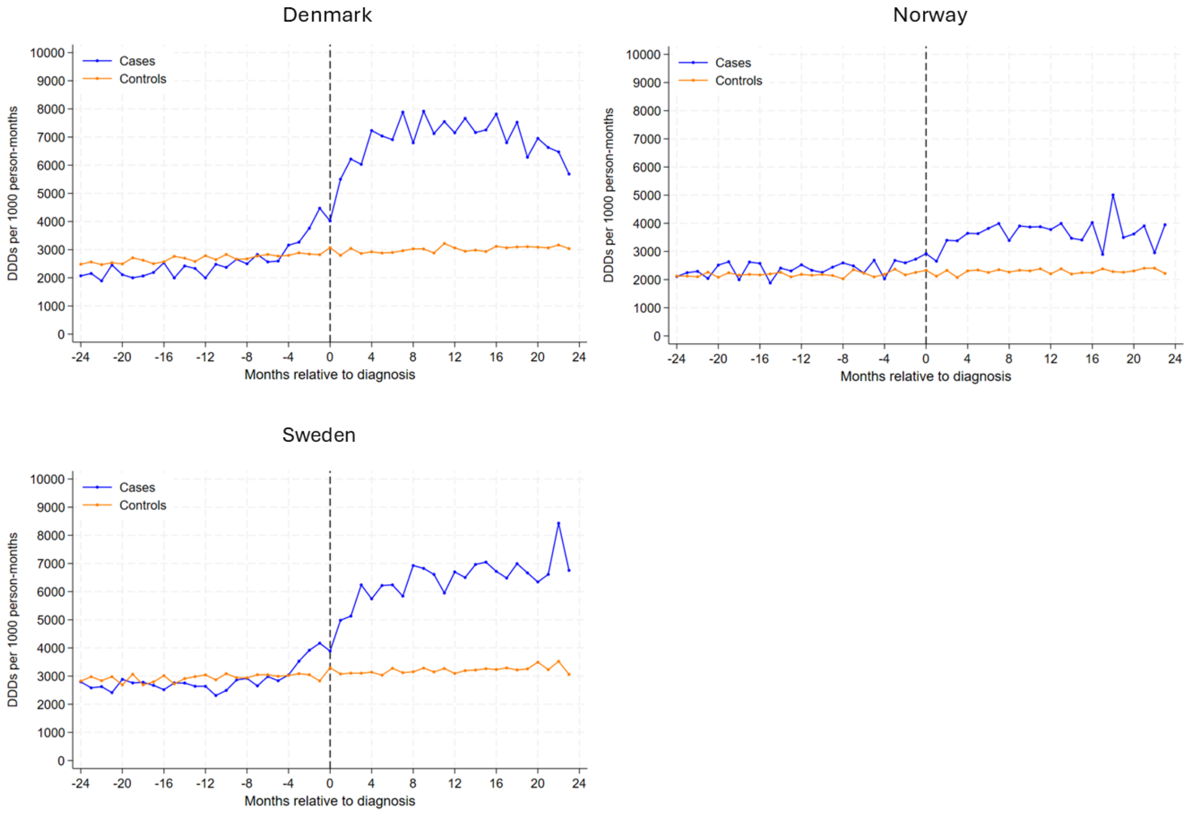

Supplementary Figure 7 Rates of new psychotropic prescriptions in 1-month intervals before and after the month of glioma diagnosis among glioma patients and the age and sex matched comparison cohort by study site restricted to 2008-2016.

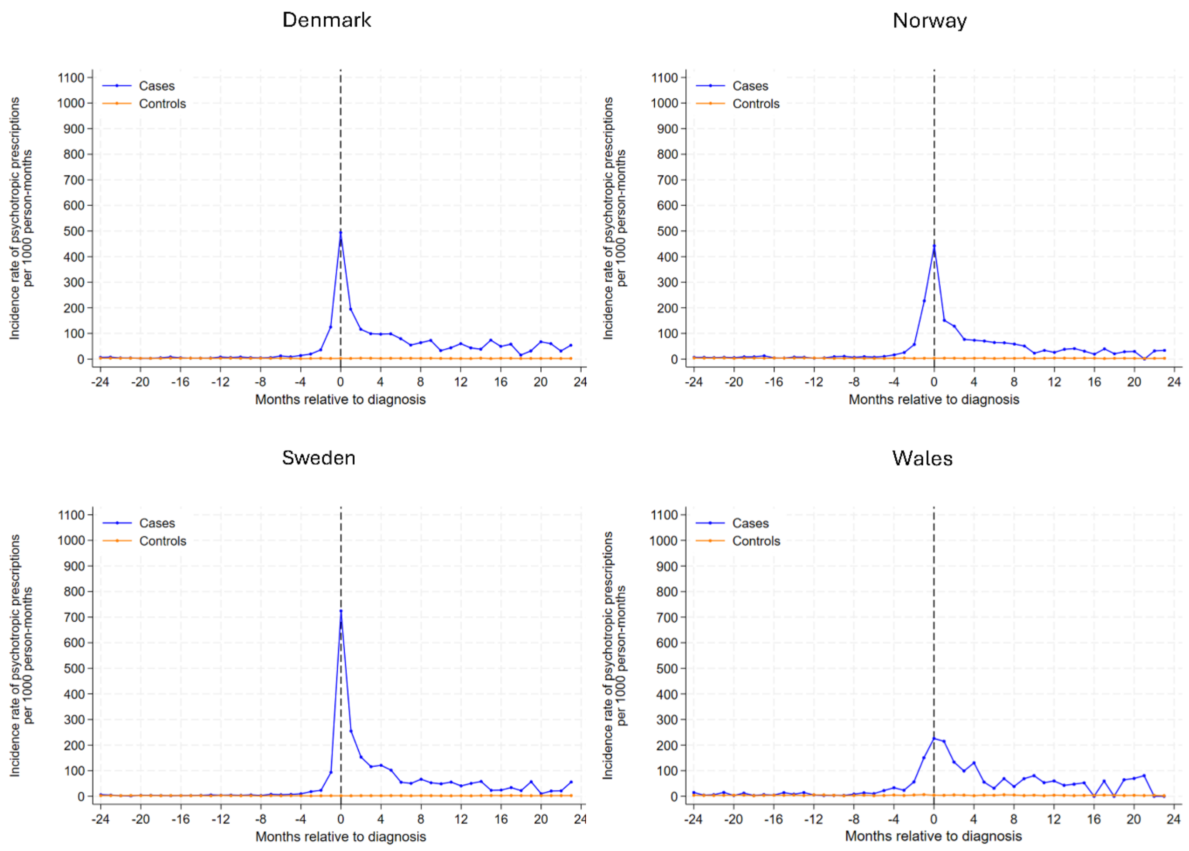

Supplementary Figure 8 Rates of all prescriptions for psychotropic medication in 1-month intervals before and after month of glioma diagnosis among glioma patients or among age and sex matched comparison cohort by study site restricted to 2008-2016.

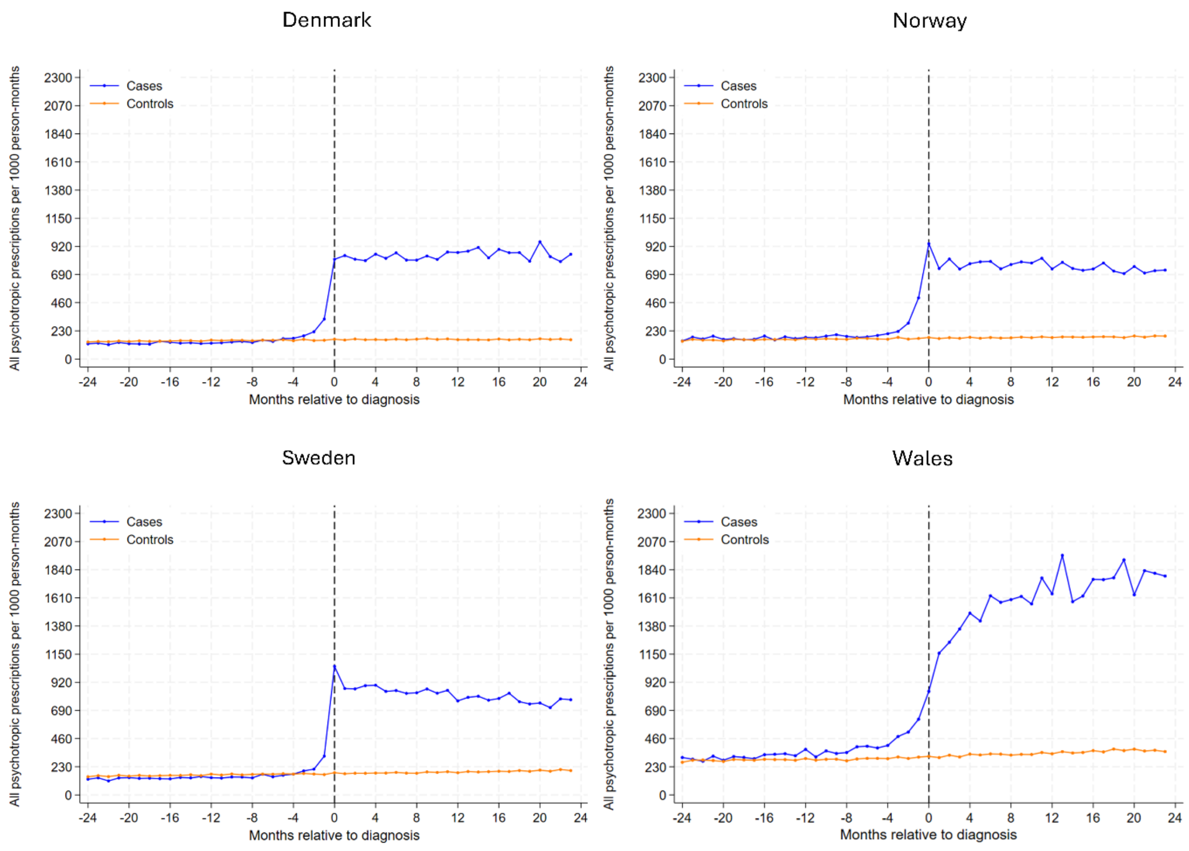

Supplement: Supplementary file 1 — Supplementary file1 (PDF 4909 KB) [file 11060_2025_4996_MOESM1_ESM.pdf]
